# A multigene and morphological analysis expands the diversity of the seabod shrimp *Xiphopenaeus* Smith, 1869 (Decapoda: Penaeidae), with descriptions of two new species

**DOI:** 10.1038/s41598-019-51484-3

**Published:** 2019-10-25

**Authors:** Abner Carvalho-Batista, Mariana Terossi, Fernando J. Zara, Fernando L. Mantelatto, Rogerio C. Costa

**Affiliations:** 1 Laboratório de Biologia de Camarões Marinhos e de Água Doce (LABCAM), Departamento de Ciências Biológicas, Faculdade de Ciências, Universidade Estadual Paulista (UNESP), Av. Luiz Edmundo Carrijo Coube 14-01, 17033-360 Bauru, SP Brazil; 2 Laboratório de Carcinologia, Departamento de Zoologia, Instituto de Biociências, Universidade Federal do Rio Grande do Sul (UFRGS), Av. Bento Gonçalves, 9500, Agronomia, 91501-970 Porto Alegre, Rio Grande do Sul Brazil; 3 Laboratório de Morfologia de Invertebrados (IML), Departamento de Biologia Aplicada, CAUNESP and IEAMar, Universidade Estadual Paulista (UNESP), FCAV. Via de Acesso Prof. Paulo Donato Castellane km 05, 14884-900 Jaboticabal, SP Brazil; 4 Laboratório de Bioecologia e Sistemática de Crustáceos (LBSC), Departamento de Biologia, Faculdade de Filosofia, Ciências e Letras de Ribeirão Preto (FFCLRP), Universidade de São Paulo (USP), Av. Bandeirantes 3900, 14040-901 Ribeirão Preto, SP Brazil

**Keywords:** Sustainability, Biodiversity

## Abstract

After being stable for nearly a century, the taxonomic history of the genus *Xiphopenaeus* has been marked by many changes in the last three decades. The taxonomic status of the Atlantic species has a low resolution, and many species are still undefined and grouped as cryptic species. Here we employed an integrative approach to define the species of *Xiphopenaeus* and the morphological characters needed to differentiate them. We combined the analyses of two molecular markers (COI and 16 S rDNA), scanning electron microscopy and light microscopy. Based on specimens from 17 localities from the Atlantic and Pacific oceans, we detected five divergent genetic groups, three in the Atlantic (A1, A2, A3) and two in the Pacific (P1, P2). Male secondary sexual characters were able to differentiate four out of the five genetic groups. Group A1 corresponds to *X. kroyeri*, and A2 and A3 correspond to new species. We redescribed the genus and two new species are described and illustrated: *Xiphopenaeus dincao* nov. sp. (A2) and *Xiphopenaeus baueri* nov. sp. (A3). Since the holotype of *X. riveti* was missing and the specimen analysed from group P2 was a female, the status of the species of *Xiphopenaeus* from the Pacific remains unresolved.

## Introduction

Cryptic species are two or more species considered as one due to their highly similar morphology^1^. The discovery of cryptic species has been increasing in the last decades due to the development of molecular tools, which indicates that they might be more common in the animal kingdom than previously thought^2,3^. An example of an unresolved taxonomic issue involving cryptic species occurs in penaeid shrimps of genus *Xiphopenaeus* Smith, 1869. This genus was described in 1869 based on the type-species *Xiphopeneus hartii* Smith, 1869, whose individuals came from the south of Bahia, Brazil. Later, *X. hartii* became a junior synonym of *Penaeus kroyeri* Heller, 1862 from Rio de Janeiro, and named *Xiphopeneus kroyeri* (Heller, 1862). For nearly 40 years *X*. *kroyeri* was the only known species of the genus. However, in 1907, a new species was described from the coast of Peru, *Xiphopeneus riveti* Bouvier, 1907^4^. In 1969, the International Commission on Zoological Nomenclature (Opinion 864) changed the genus spelling to *Xiphopenaeus*^5^.

*Xiphopenaeus kroyeri* has an Atlantic distribution from North Carolina (USA) down to Rio Grande do Sul (Brazil). *Xiphopenaeus riveti* occurs in the Pacific from Sinaloa (Mexico) to Paita (Peru)^4^. Due to the high morphological similarity between *X. kroyeri* and *X. riveti* their taxonomy remained controversial: *X. riveti* was considered as a variety of *X. kroyeri*, or its sister species^6,7^. Perez-Farfante & Kensley^4^ revised the suborder Dendrobranchiata and considered *X. riveti* as a junior synonym of *X. kroyeri*. Thus, the genus was again considered monotypic and *X. kroyeri* its only valid species^8^. Gusmão *et al*.^9^ addressed the issue again, using molecular data from PCR/RFLP, isoenzyme polymorphisms and Cytochrome C Oxidase Subunit I sequences. They proposed the revalidation of *X. riveti* from the Pacific plus the existence of cryptic speciation in *Xiphopenaeus kroyeri*, which contained two entities they call: *Xiphopenaeus* sp. 1 and *Xiphopenaeus* sp. 2. These entities were not formally described, and neither were the morphological characters that could differentiate the species (synapomorphies). Eight years later, Piergiorge *et al*.^10^ came to the same conclusions, also using molecular techniques, but once again, no definitive morphological characters were given to differentiate these entities. Recently, Kerkhove *et al*.^11^, have detected *Xiphopenaeus* sp. 2 in north part of South America (Guiana and Caribbean Ecoregions), and pointed out differences in the colour of individuals between the two species.

*Xiphopenaeus kroyeri* sensu lato represents ~40% of the total shrimp fisheries in the Brazilian coast^12^ and often accounts for 90% of the shrimp biomass captured in shallow waters, up to 20 m of depth^13–15^. Moreover, the species is the second most important fishery resource of the southeast of Brazil, and the most targeted shrimp in the State of São Paulo, were the catch reach the maximum in early 1980s (8,905 tons) and then start to decrease until 2000 (629 tons), since then the biggest catch occurred in 2012 (3,258 tons)^16–18^. However, in 2018 *X*. *kroyeri* becomes the most exploited fishery resource in this state (2,246 tons)^19^.

Naturally, given the commercial importance of this resource, *Xiphopenaeus kroyeri* sensu lato has been constantly studied, despite the lack of a definitive taxonomic solution to define and/or distinguish the entities that are known to exist (c.f. Gusmão *et al*.^9^, Piergiorge *et al*.^10^). In the last 13 years, more than 50 articles have been published on diverse subjects — population biology, ecology, fishery biology, physiology, toxicology, sperm ultrastructure, bycatch — considering *X. kroyeri* as the only species of *Xiphopenaeus* in the Atlantic. Certainly, this was caused by the lack of knowledge on the morphological characters that could be used to differentiate the two species.

The correct taxonomic identification of important fishery resources is essential to enhance biodiversity knowledge and to develop adequate management and conservation strategies^20–22^. Recently, the combination of molecular tools and the detailed morphological analyses of the reproductive structures has proved to be very effective to solve taxonomic problems and to differentiate and describe cryptic species of decapod crustaceans^23,24^. Therefore, considering the controversial taxonomic history of *Xiphopenaeus* explained above and its high economic importance, here we employed an integrative analysis and combined molecular and morphological tools to characterize the species that constitute the genus *Xiphopenaeus*, based on specimens from 17 localities, 15 in the Atlantic and two in the Pacific cost of America. We provide a detailed redescription of the genus and the description of two new species.

## Results

### Molecular analysis

About 1,125 base pairs (bp), from our double-genes analyses, were used to construct the phylograms. A total of 91 sequences of the COI gene (barcoding region) were obtained. The final alignment had 596 base pairs. There were 111 variable sites: 11 in the 1^st^ codon base, 2 in the 2^nd^, and 96 in the 3^rd^; and 93 variable sites were phylogenetically informative (Supplementary Fig. 1). The average nucleotide composition was: T = 33.7%, C = 20.8%, A = 27.4%, G = 18.1%. We obtained 15 sequences of the 16S gene. The final alignment had 529 base pairs. We detected 25 variable sites of which 16 were phylogenetically informative (Supplementary Fig. 2). An average nucleotide composition was: T = 31.0%, C = 21.6%, A = 34.7%, G = 12.8%. After adding the sequences of the four external group species, we obtained 179 variable sites, of which 81 were phylogenetically informative.

### Phylogenetic analyses

Maximum likelihood phylogram based on the COI (barcoding region) gene indicated the existence of five groups, three from the Atlantic Ocean (A1, A2 and A3) and two from the Pacific (P1 and P2).

**Figure 1:**
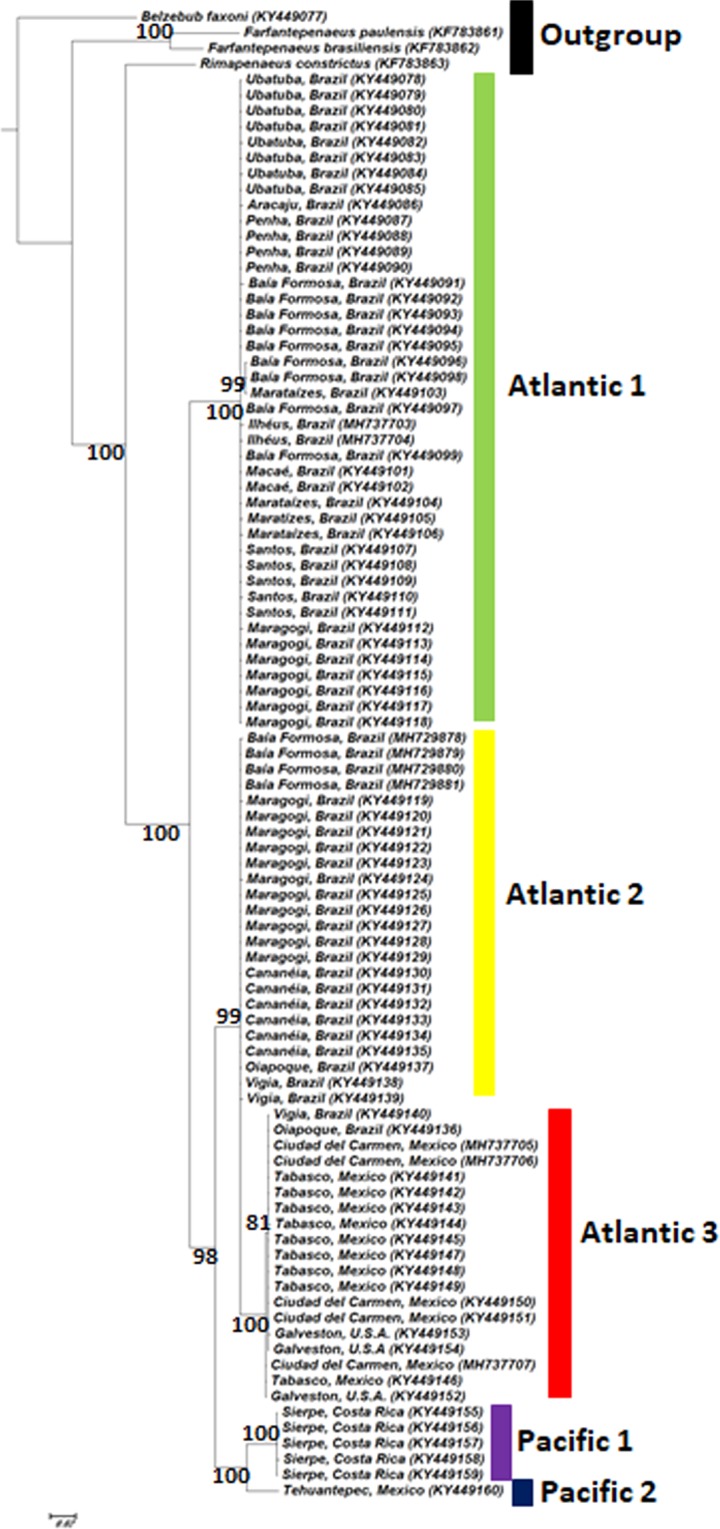
Maximum likelihood phylogram of 91 specimens of *Xiphopenaeus*, based on the COI gene (barcoding region). The numbers near the branches are bootstrap values (values lower than 50 are not shown).

**Figure 2:**
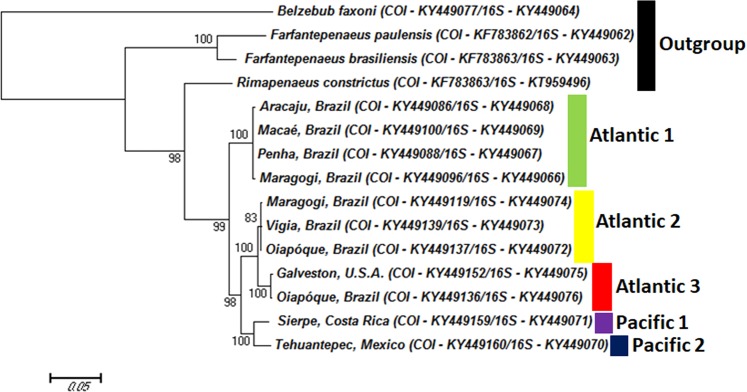
Maximum likelihood phylogram of 16 specimens of *Xiphopenaeus*, based on the concatenated and partitioned sequences of the genes COI (barcoding region) and 16S. The numbers near the branches are bootstrap values (values lower than 50 are not shown).

The phylogram of the concatenated COI and 16S sequences supported the same division into five groups (Figs. 1 and 2). Both trees show that P1 and P2 form a well-supported clade, which is a sister group of the clade formed by A2 and A3, while A1 forms an external clade regarding A2 and A3.

**Figure 3:**
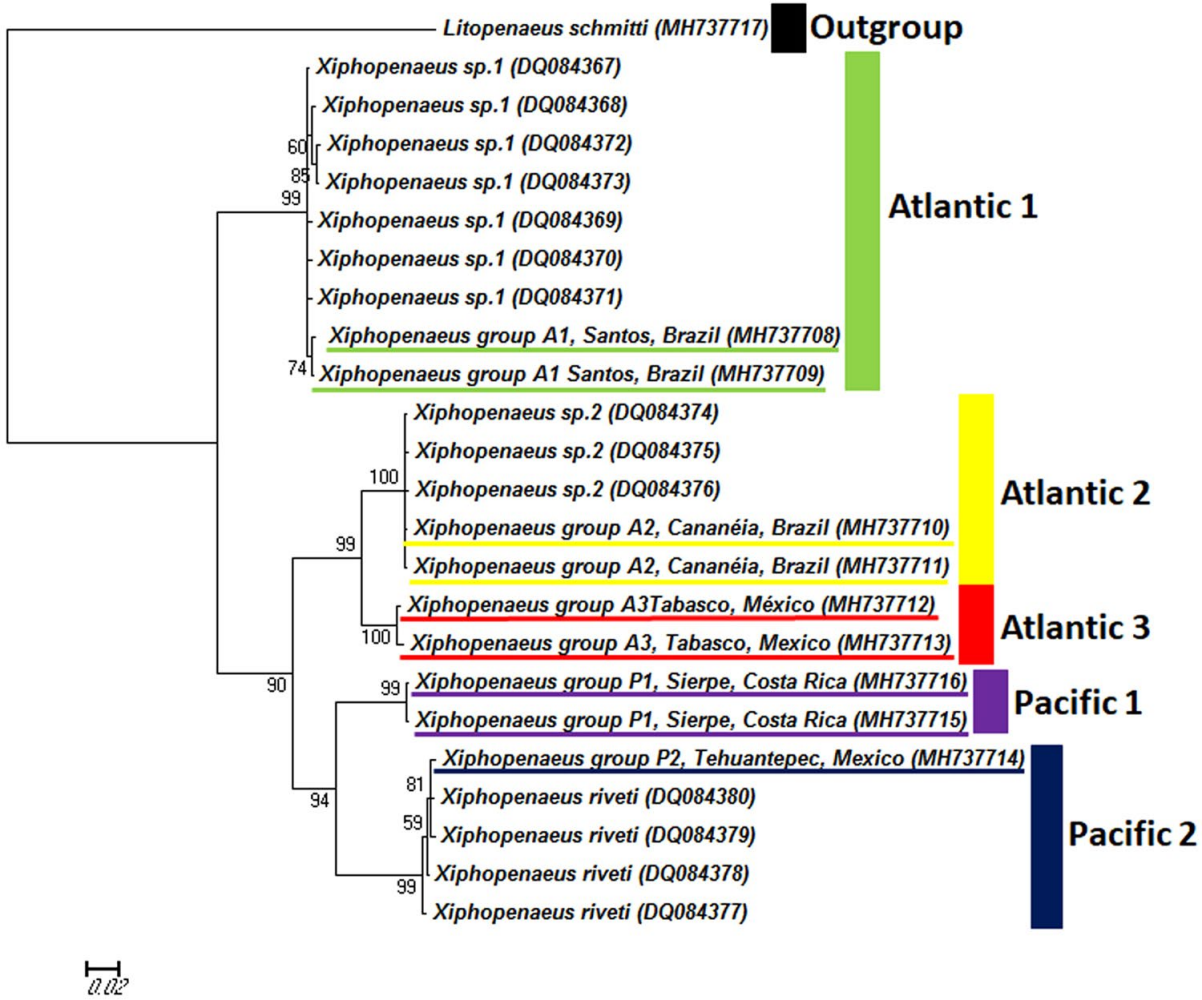
Maximum likelihood phylogram of specimens of *Xiphopenaeus* based on the COI gene (Palumbi region), including the specimens from our study assigned to the five genetic groups (underlined) and the sequences from Gusmão *et al*. (2006).

The comparison of our specimens with those of Gusmão *et al*.^9^, based on the Palumbi region of the COI gene^25^, indicate that our clade A1 corresponds to *Xiphopenaeus* sp1, and clade A2 corresponds to *Xiphopenaeus* sp 2. Clade P2 corresponds to *Xiphopenaeus riveti*, and clades A3 and P1 are two genetic groups previously undetected (Fig. 3).

### Genetic distances

**Figure 4:**
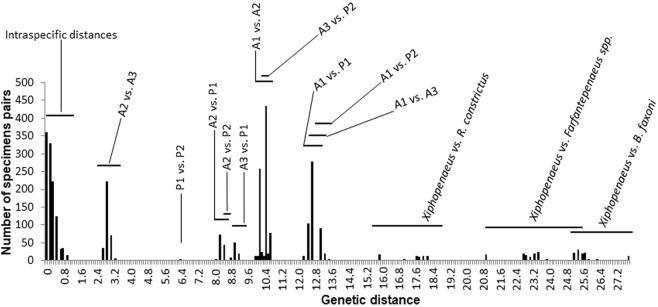
Histogram of the pairwise genetic distances (Kimura-2-parameters) for the COI gene (barcoding region), within *Xiphopenaeus*, and between *Xiphopenaeus* and the outgroups. The lines above the bars indicate the range of values found in the pairwise comparisons between the groups revealed in the previous analyses. A1: Atlantic 1, A2: Atlantic 2, A3: Atlantic 3, P1: Pacific 1, P2: Pacific 2.

**Figure 5:**
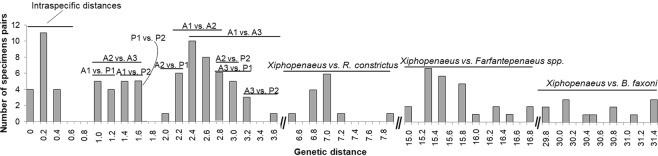
Histogram of the pairwise genetic distances (Kimura-2-parameters) for the 16S gene (barcoding region), within *Xiphopenaeus*, and between *Xiphopenaeus* and the outgroups. The lines above the bars indicate the range of values found in the pairwise comparisons between the groups revealed in the previous analyses. A1: Atlantic 1, A2: Atlantic 2, A3: Atlantic 3, P1: Pacific 1, P2: Pacific 2.

The genetic distance within *Xiphopenaeus* regarding the COI gene varied from 0 to 13.5%, and the distance to the outgroups was 15.7−27.8%. There was a clear separation between groups, i.e., there was a low genetic distance within groups (0−1%) and a higher distance between groups. The lowest between-group distances were between A2 and A3, 2.7−3.3%, and the highest were 12.9−13.5%, between A1 and P2 (Fig. 4). Regarding the 16S gene, the genetic distance within *Xiphopenaeus* varied from 0.0 to 2.9%, and the distance to the outgroups varied from 7.3 to 38.1%. The results of the 16S corroborated the groups suggested by the COI, and the within-group distances were 0.0−0.4%. The lowest between-group distances occurred between A1 and P1 (1.0–1.2%), and the highest, between A3 and P2 (3.2–3.6%) (Fig. 5). Thus, based on COI and 16S genes, there is evidence of at least five genetic groups in *Xiphopenaeus*. Based on the combined analyses with morphological characters, two of these groups will be described below as new taxonomic entities.

### Morphological assessment

#### Gross Morphology

In the gross morphological analyses, we used 79 individuals from 16 localities which had at least one of the genes sequenced. The comparative analysis of the carapace (spine, grooves, and carinas), legs and mouthparts did not point to substantial variation capable to differentiate the genetic groups. The number of rostral teeth varied from four to six, but most individuals had five, irrespectively of the group. Individuals bearing four were seen only in groups P1 and A3, while six teeth were seen only in groups A1 and A2. There was individual variation regarding the teeth shape, rostrum length (in relation to the carapace), groove and carina depth, but they did not lead to a separation of the groups. The presence and size of the teeth of the dorsomedial carina in the fourth, fifth, and sixth abdominal segments also varied. Although in A1 they were smaller and less frequent, this character was also not enough to separate the groups.

The petasma and *appendix masculina*, however, were much more informative and separated the individuals according to the genetic groups. Thus, we decided to update the general description of *Xiphopenaeus* given by Perez-Farfante & Kensley^4^ adding a detailed redescription of these two male structures. The description of the *appendix masculina* is given for the first time on the literature on *Xiphopenaeus*.

### Systematics

Suborder Dendrobranchiata Spence Bate, 1888

Superfamily Penaeoidea Rafinesque-Schmaltz, 1815

Family Penaeidae Rafinesque-Schmaltz, 1815

*Xiphopenaeus* Smith, 1869

*Penaeus* – Heller, 1862, Sber. Akad. Wiss. Wien, 45(1): 425.

*Xiphopeneus* Smith, 1869, Trans. Conn. Acad. Arts Sci., 2(1): 27. For details see Perez-Farfante & Kensley (1997), p. 149–152.

### Redescription of the genus *Xiphopenaeus*

Integument glabrous. Rostrum long, considerably overreaching antennular peduncle, usually longer than carapace in adults, sinuous, styliform anteriorly; armed with dorsal teeth only, situated basally; epigastric tooth distinctly separated from first rostral. Carapace with orbital angle well marked, antennal, and hepatic spines present; pterygostomian angle produced but lacking spine; postocular sulcus well marked; clearly distinct orbito-antennal sulcus; short, almost indistinct cervical sulcus, clearly distinct orbito-antennal sulcus; hepatic sulcus and sharp hepatic carina reaching only base of pterygostomian region and posteriorly merging with long branchiocardiac sulcus and carina, respectively; longitudinal suture extending to about mid length of carapace, transverse suture lacking (in adults). Abdomen with sixth somite bearing interrupted cicatrix. Telson unarmed. Antennule lacking parapenaeid spine; antennular flagella long, dorsal (twice or more as long as carapace) longer than ventral. Palp of first maxilla entire, gradually tapering distally, produced into small, triangular, setose proximolateral lobule, broad, setose proximomesial and quite small, acute setose distomesial lobules, latter armed with slender spine; distolateral row of small spines present on ventral surface. Fourth and fifth pereopods long, much longer than third, subflagelliform, each with multiarticulate dactyl. Basial and ischial spines on first pereopod only.

Thelycum closed, with single plate of sternite XIV smooth, broad, not produced anteriorly into pair of flaps, its anterolateral hoods much reduced; anterior sternal invagination nearly as broad as sternite, forming spacious pocket extending as far as posterior thoracic ridge; median protuberance of sternite XIII also broad but quite short. Paired seminal receptacles (spermatheca) is bilobed, each consisting of large posterior lobe and small anterolateral one; sinuous slit-like opening lying on anterior end of posterior lobe, connecting with median pocket and extending laterally dorsal to posterolateral extremity of median protuberance.

### General description of petasma and *appendix masculina* of the genus *Xiphopenaeus*

**Figure 6:**
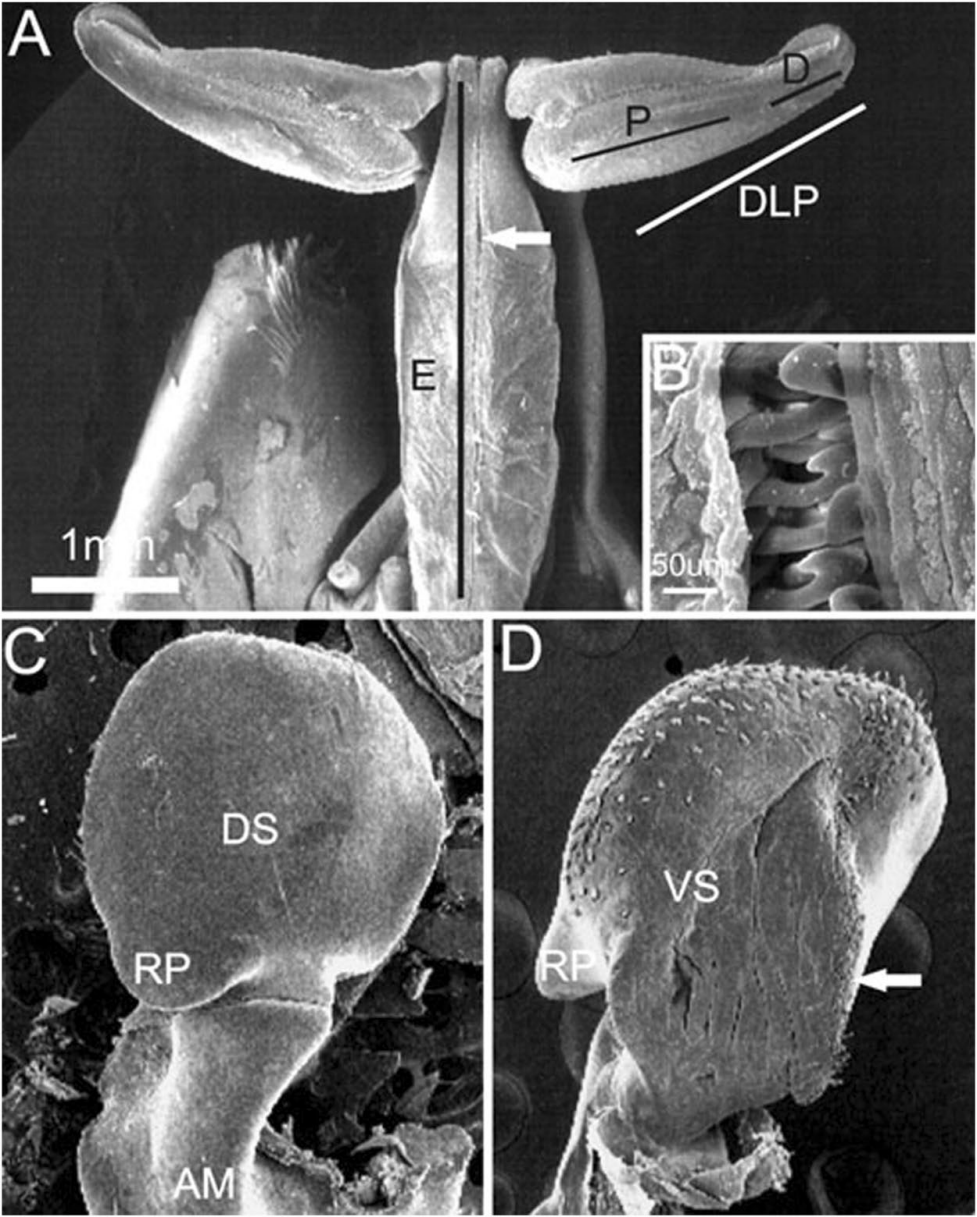
Scanning electron microphotographs showing the general morphology of the secondary sexual characters of *Xiphopenaeus*. A, *Xiphopenaeus dincao* nov. sp. (CCDB 6499): petasma (E - endopod; DLP - distolateral projection; P - proximal region of the DLP; D - distal region of the DLP); B, *Xiphopenaeus dincao* nov. sp. (CCDB 6499): junction between the endopods with cincinuli (seta); C, *Xiphopenaeus kroyeri* (CCDB 5019): *appendix masculina* in dorsal view (DS – dorsal surface; RP – rounded projection); D, *Xiphopenaeus kroyeri* (CCDB 5019): *appendix masculina* in ventral view (VS - ventral surface; RP - rounded projection; white arrow indicating the row of spines of the posterior margin).

Petasma formed by the union of the endopods of the first pleopod pair, joining the two dorsolateral lobes by the cincinuli (Fig. 6B), and bearing a horn-like distolateral projection (DLP) (Fig. 6A). The distolateral projection has two regions. The proximal region extends from the endopod junction until 2/3 or 3/4 of the distolateral projection length (depending on the species). The distal region, which is oblique to the proximal, bears the opening; its posterior surface is covered by a row of teeth whose morphology varies in each species (Fig. 6A).

The *appendix* *masculina* is subcircular (Fig. 6C) and has a rounded projection whose posterior margin that varies in length and is oriented towards the body midline. The dorsal surface is smooth (Fig. 6C); the central region of the ventral face is concave or convex and has rows of spines with varied distribution (Fig. 6D). Both characters vary depending on the species, and the species-specific descriptions are given below.

**Figure 7:**
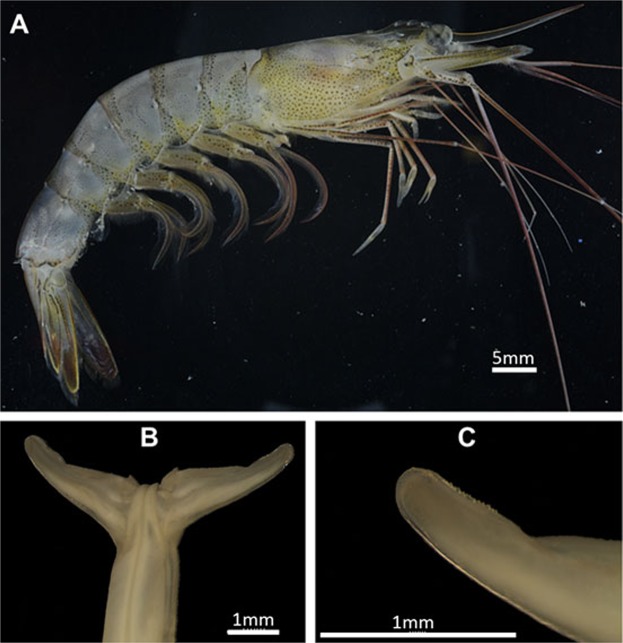
*Xiphopenaeus kroyeri* (Heller, 1862) (**A**) Lateral view (specimen ULLZ 15974); male; Ubatuba, São Paulo, Brazil. Photo: Darryl L. Felder; (**B**) Dorsal view of petasma (specimen CCDB 5019); (**C**) Detail of the right distolateral projection (specimen CCDB 5019).

**Figure 8:**
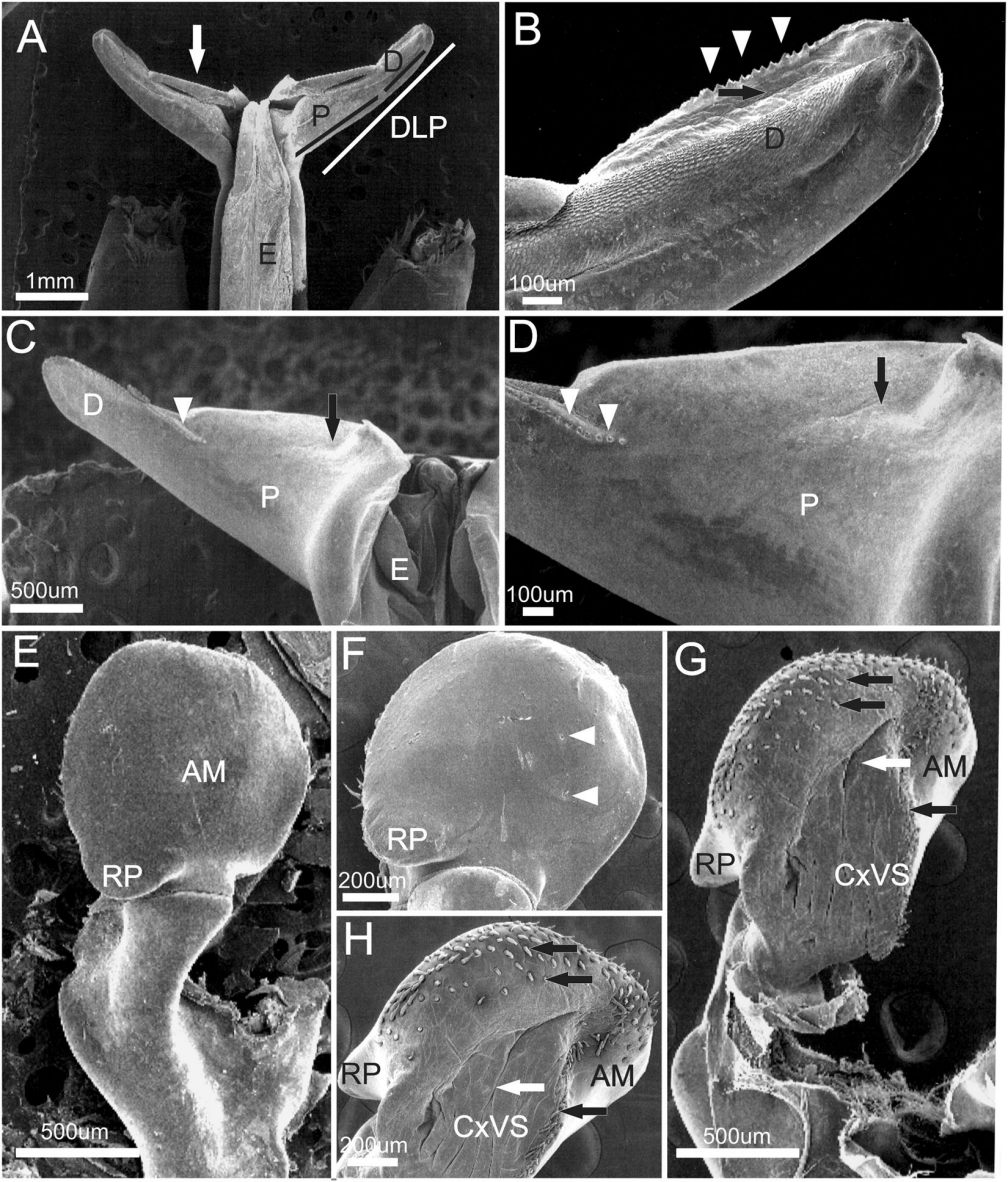
Scanning electron microscopy. Brazil: São Paulo, Ubatuba. 4♂ (CCDB 5019). Male secondary sexual characters of *Xiphopenaeus kroyeri*. (**A**) petasma in dorsal view; the white arrow indicates the posterior margin of the distolateral projection; (**B**) distal region of the distolateral projection; white arrowheads indicate the teeth; black arrows indicate the petasma opening; (**C**) distolateral projection in ventral view; white arrowheads indicate the row of teeth of the distal region of the distolateral projection; the black arrow indicates the carina of the proximal region of the distolateral projection; (**D**) detailed ventral view of distolateral projection; white arrowheads indicate the row of teeth of the distal region of the distolateral projection; the black arrow indicates the carina of the proximal region of the distolateral projection; (**E**) *Appendix masculina* in dorsal view; (**F**) *Appendix masculina* in dorsal view; white arrowheads indicate the spines; (**G**) *Appendix masculina* in ventral view; the black arrow indicates the row of spines of the posterior margin, white arrows indicate the convex central region; (**H**) *Appendix masculina* in ventral view; the black arrow indicates the row of spines of the posterior margin, white arrows indicate the convex central region.

**Figure 9:**
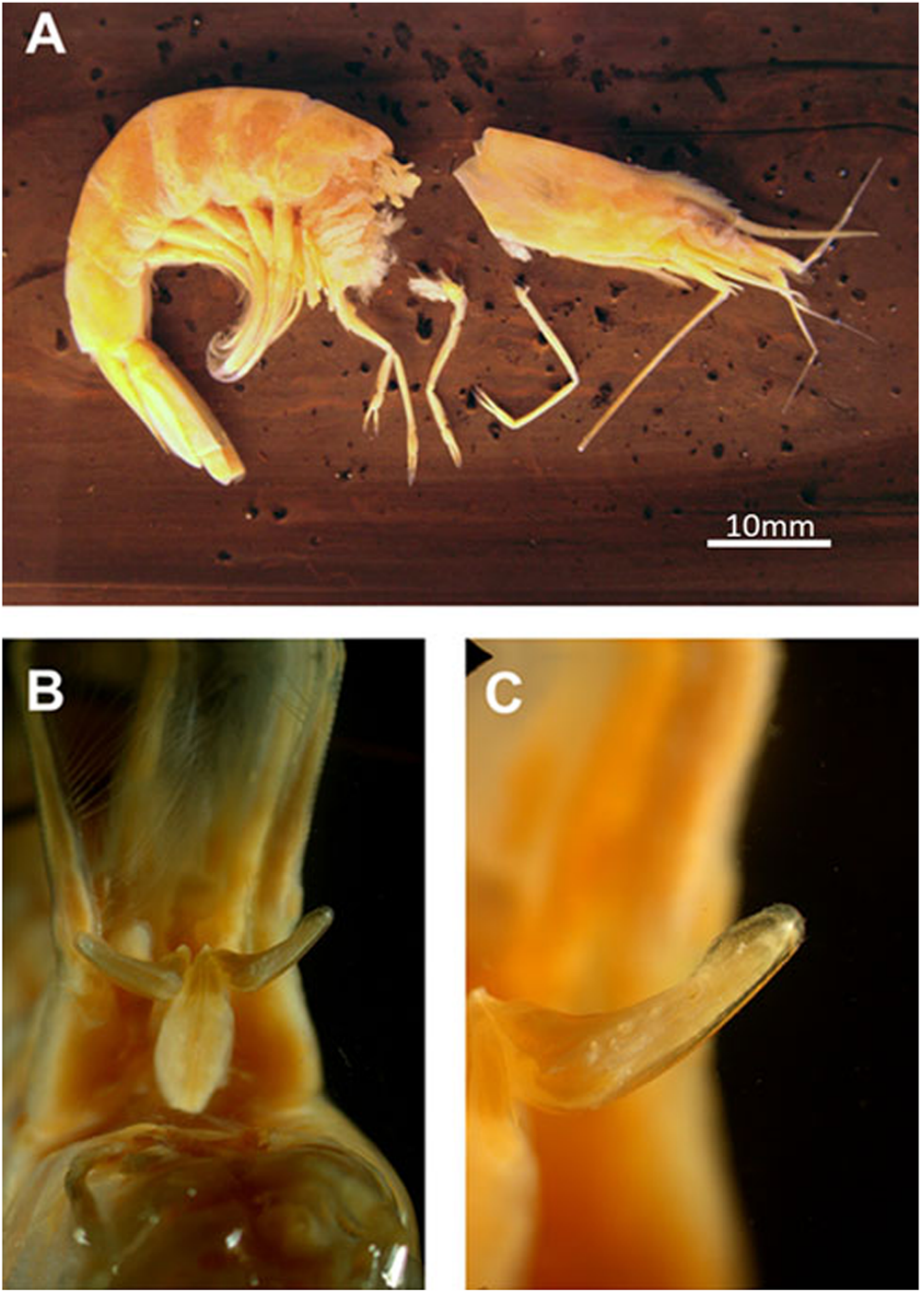
Holotype of *Xiphopenaeus kroyeri* (Heller, 1862): NHMW 342, Rio de Janeiro, Brazil collected by Kröyer, male. (**A**) lateral view; (**B**) Petasma in dorsal view; (**C**) detail of the right distolateral projection. Photos by Peter Dworschak.

***Xiphopenaeus kroyer****i* (Heller, 1862) (Figs. 7–9).

*Peneus Kroyeri* Heller, 1862b: 425; Plate 2, fig. 51. [Rio Janeiro]

*Xiphopeneus hartii* Smith, 1869: 27, 40; Plate 1, Fig. 1. [Caravelas, Estado da Bahia, Brazil]

#### Material examined

Holotype: Rio de Janeiro, 1♂, col. Kröyer, NHMW 342 (Fig. 9).

#### Additional material

Brazil: Rio Grande do Norte, Baía Formosa, 06°21′23,3″S − 35°00′24,7″W, 25/IV/2014, col. M. Lopes & A. Carvalho-Batista, 6♂, 3♀ (CCDB 5337) – Alagoas, Maragogi, Praia de Maragogi, 09°00′48,59″S − 35°13′14,46″W, 05/X/2013, col. F.L. Mantelatto & F.B. Mantelatto, 1♂, 3♀ (CCDB 5338) - Sergipe, Aracajú, Praia do Atalaia, 26/VII/2013, col. G.L. Hirose, 1♀ (CCDB 5246) – Espírito Santo, Marataízes, 20°59′S − 40°47′W, 20/VI/2012, Col. F.L. Carvalho, D. Peiró & R. Robles, 3♂, 1♀ (CCDB 3985) – Rio de Janeiro, Macaé, 22°23,44′S − 41°44,57″W, 21/VII/2014, R.C. Costa *et al*., 3♂ (CCDB 5339) – São Paulo, Ubatuba, Praia do Cedro, 23°32′38,4″S − 45°09′54″W, 22/VII/2013, R.C. Costa *et al*., 4♂ (CCDB 5019) – Santos, 24°04′55,″S − 46°16′56,8″W, 24/X/2011, R.C. Costa *et al*., 2♂, 2♀ (CCDB 3663) – Santa Catarina, Penha, V/2014, R.C. Costa *et al*., 3♂, 1♀ (CCDB 5292).

#### Morphological characterization to be used in comparisons with other species

Petasma: In dorsal view, the posterior margin of the proximal region of the distolateral projection is straight (Figs. 7B, 8A). The distal region extends through 1/3 of the distolateral projection length and forms an obtuse angle towards the central-posterior part of the petasma (Figs. 7B,C, 8A,B). The opening is narrow and long and crevice-like and bears a row of upright teeth in the posterior margin, which is straight (Figs. 7C, 8B). In ventral view, the row of teeth of the distal region posterior margin forms a carina; the part of the carina next to the endopod is more evident (Fig. 8C,D).

*Appendix masculina* subcircular with a rounded projection towards the body median part and bearing two spines in the dorsal surface (Fig. 8E,F). The posterior margin of the ventral surface is covered by small sparse spines, with a few rows in the central convex region (Fig. 8G,H).

#### Type locality

Rio de Janeiro, State of Rio de Janeiro, Brazil

#### Distribution

Colombia, Venezuela, Guyana, Suriname, French Guyana, Brazil (Maranhão, Rio Grande do Norte, Alagoas, Sergipe, Bahia, Espírito Santo, Rio de Janeiro, São Paulo, Santa Catarina).

#### Remarks

The comparison with the holotype of *Xiphopenaeus* *kroyeri*, deposited in the Museum of Natural History of Vienna (Austria), indicated that this species corresponds to our clade A1 and also to *Xiphopenaeus* sp.1 detected by Gusmão *et al*.^9^ Based on the material examined here, this species seems to be the very abundant. It was the most abundant species in almost all localities of the northern, southern, and southeastern coasts of Brazil.

### *Xiphopenaeus dincao* nov. sp

( Figs.10 and 11).

**Figure 10:**
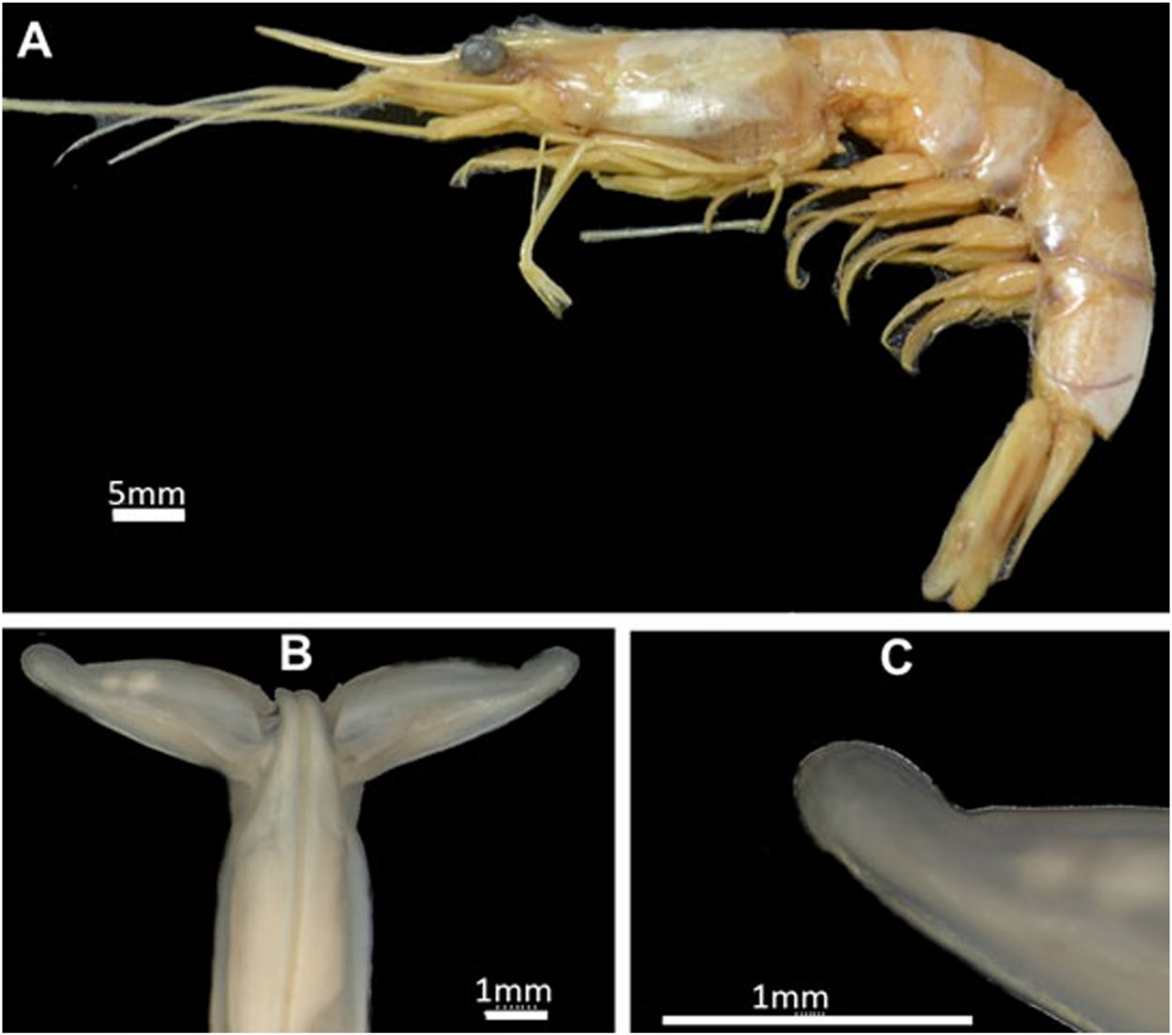
*Xiphopenaeus dincao* nov. sp. (**A**) Lateral view of the holotype (MZUSP 39350), male, Maragogi, Alagoas, Brazil. (**B**) Petasma in dorsal view; (**C)**. Detail of the right distolateral projection. Photos by Julia Fernandes Perroca.

**Figure 11:**
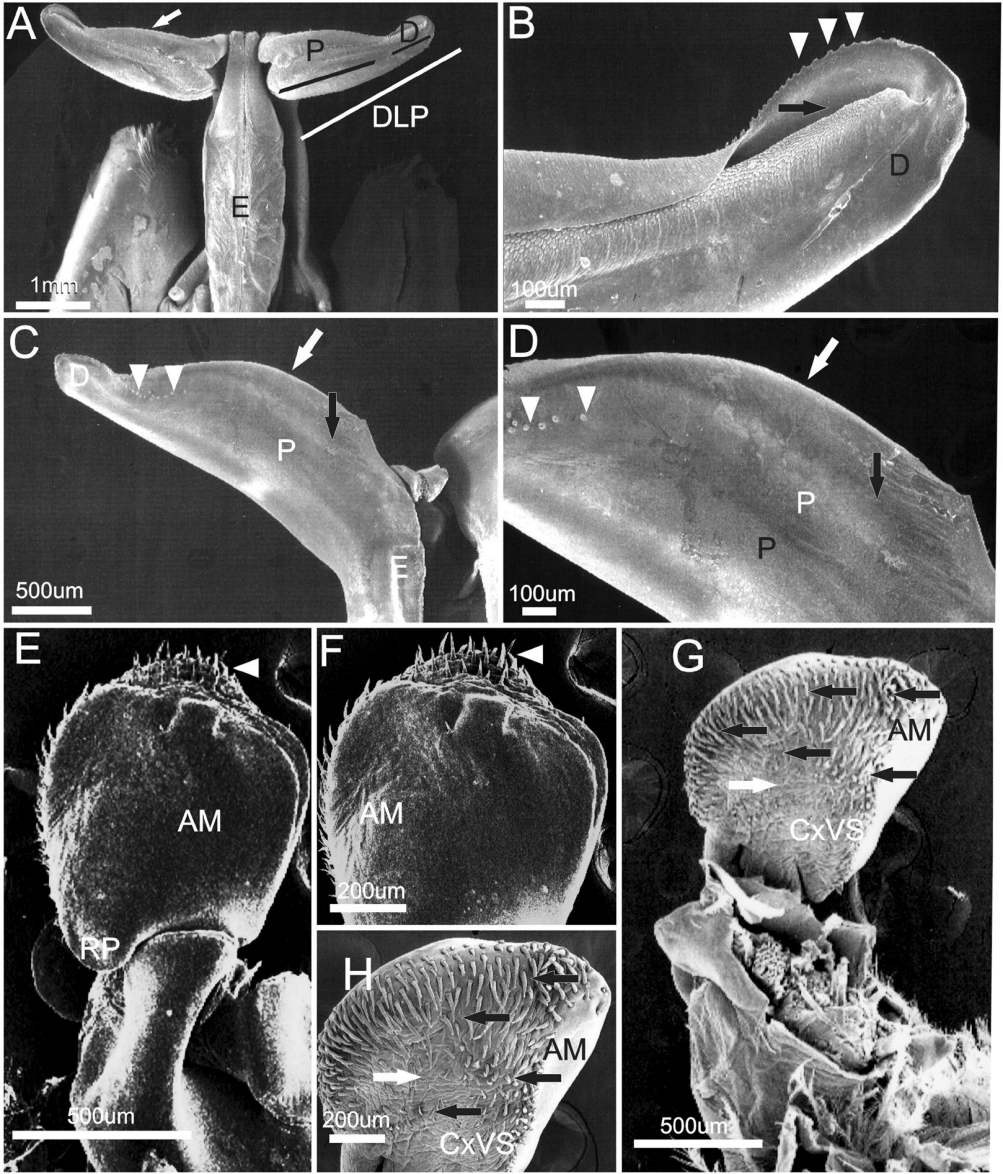
Scanning electron microscopy. Brazil: Alagoas, Maragogi, 3♂ (CCDB 6499), 1♂ (MZUSP 39350). Male secondary sexual characters of *Xiphopenaeus dincao* nov. sp. (**A**) petasma in dorsal view; the white arrow indicates the posterior margin of the distolateral projection; (**B**) distal region of the distolateral projection; white arrowheads indicate the teeth; black arrows indicate the petasma opening; (**C**) distolateral projection in ventral view; white arrowheads indicate the row of teeth of the distal region of the distolateral projection; the black arrow indicates that the carina of the proximal region of the distolateral projection is absent; (**D**). Detailed ventral view of distolateral projection; white arrowheads indicate the row of teeth of the distal region of the distolateral projection; the black arrow indicates the carina of the proximal region of the distolateral projection is absent; (**E**) – *Appendix masculina* in dorsal view; (**F**) *Appendix masculina* in dorsal view; white arrowheads indicate the spines; (**G**) *Appendix masculina* in ventral view; the black arrow indicates the row of spines of the posterior margin, white arrows indicate the central convex region; (**H**) – *Appendix masculina* in ventral view; the black arrow indicates the row of spines of the posterior margin, white arrows indicate the central convex region.

*Xiphopenaeus* sp. 2 — Gusmão *et al*. 2006: 491, 496–500, Figs 3–5; Kerkhove *et al*. 2019: 853–858, Figs 1–5.

*Xiphopenaeus* sp. II — Piergiorge *et al*. 2014: 349–353, Figs 2–3.

#### **Holotype**

Brazil: Alagoas, Maragogi, Praia de Maragogi, 09°00′48.59″S − 35°13′14.46″W, 05/X/2013, col. F.L. Mantelatto & F.B. Mantelatto, 1♂ (MZUSP 39350).

#### **Paratypes**

Brazil: Alagoas, Maragogi, Praia de Maragogi, 09°0′48.59″S − 35°13′14.46″W, 05/X/2013, col. F.L. Mantelatto & F.B. Mantelatto, 5♂, 2♀ (CCDB 6499) - São Paulo, Cananéia, X/2014, R.C. Costa, 2♂, 5♀ (CCLC 418).

#### Additional material examined

Brazil: Amapá, Oiapoque, Estuário do Rio Oiapoque, Parna Cabo Orange, 04°22′17.6″N–51°24′26.4″W, 22/VIII/2013, col. I.M. Vieira, A.G. Santiago & E.G. Oliveira, 1♂ (IEPA 1618) – Pará, Vigia, Ponta Seca, 0°51′45.00″S – 48°7′50.00″W, 19/XI/1994, col. M.P. Barros, 2♂ (MCP 2024).

#### Description

Petasma: In dorsal view, the proximal region occupies 3/4 of the distolateral projection length (Figs. 10B, 11A), with rounded posterior margin (Figs. 10A,B, 11A,B). The distal region is as long as wide and bears a convex row of teeth and the opening, which is wide and rounded (Figs. 10C, 11B). In ventral view the row of teeth of the posterior margin enters the proximal region but ends abruptly before its wider portion. In this species the carina of the ventral surface of the proximal region is absent (Fig. 11C,D). In dorsal view the *appendix masculina* is subcircular but less rounded than in *X. kroyeri* and its protruding tip has many spine rows (Fig. 11E,F). The posterior rounded projection is longer and narrower than in *X. kroyeri*. In ventral view the spines are more prominent in the margins and become smaller over the central convex region (Fig. 11G,H).

#### Etymology

The specific epithet “dincao” is given in the honour of the late Dr. Fernando D′Incao, in recognition of his contribution to the Brazilian Carcinology, in particular to the study of penaeoid shrimps from the Brazilian coast. It is to be treated as a noun in apposition.

#### Type locality

Maragogi, Alagoas, Brazil.

#### Distribution

Colombia^11^, Suriname^11^, French Guiana^11^, Brazil^9,10^ and presen study (Amapá, Pará, Rio Grande do Norte, Alagoas, Bahia, São Paulo).

#### Remarks

This species refers to the specimens of clade A2, previously known as *Xiphopenaeus* sp. 2^9–11^. Besides the description, this is the first record of this taxon to Amapá, Pará and Alagoas. Despite the vast list of works in the literature that studied biology of *X. kroyeri*, it was not possible to identify in which of these studies were used specimens of the described new species and thus the synonymic list was short.

### *Xiphopenaeus baueri* nov. sp

(Figures 12−13).

**Figure 12:**
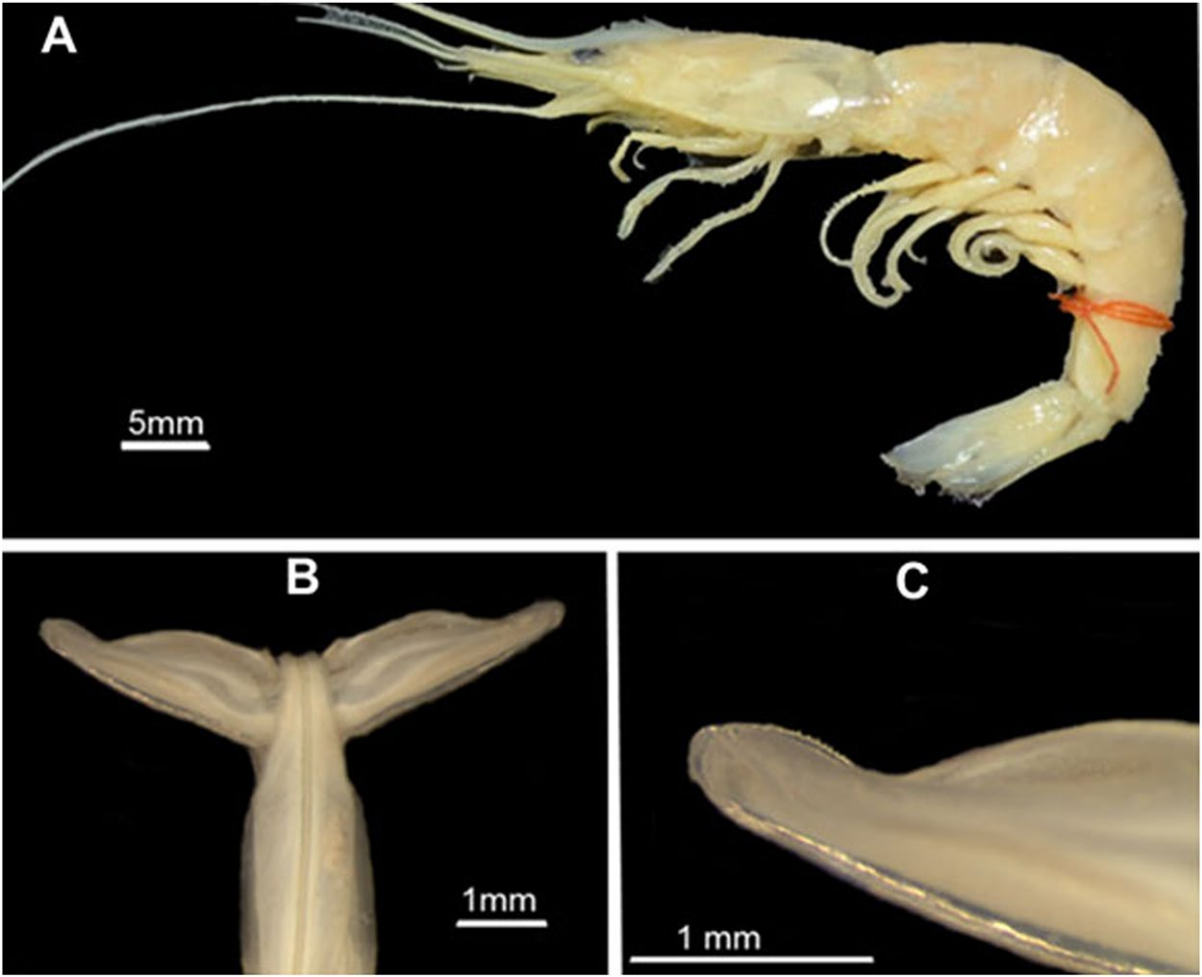
*Xiphopenaeus baueri* nov. sp.; (**A**) Lateral view of the holotype (MZUSP 39351), male, Tabasco, Mexico; (**B**) Petasma in dorsal view; (**C**) Detail of the right distolateral projection. Photo by Julia Fernandes Perroca.

**Figure 13:**
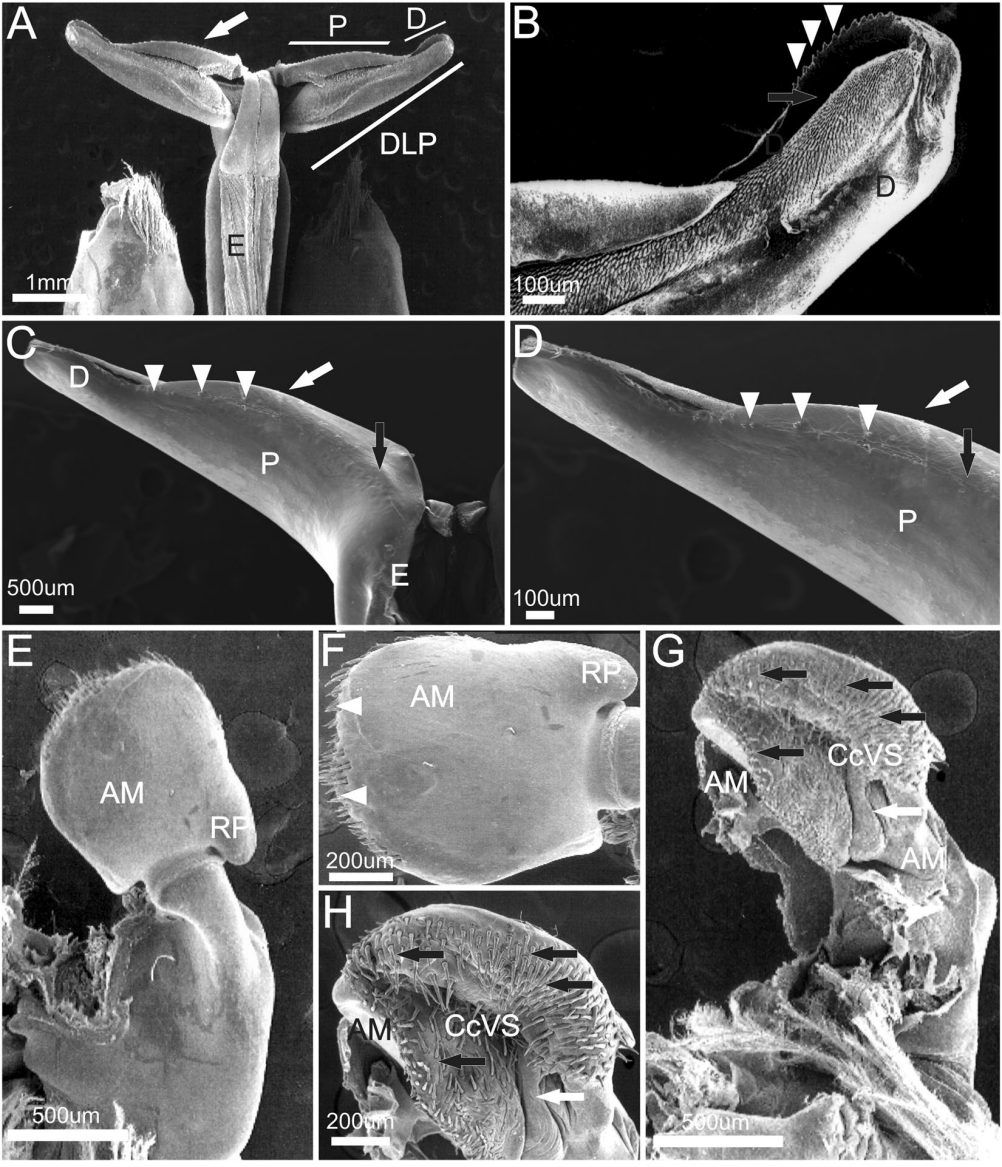
Scanning electron microscopy. Mexico: Tabasco, 3♂ (CCDB 5461), 1♂ (MZUSP39351). Male secondary sexual characters of *Xiphopenaeus baueri* nov. sp. (**A**) petasma in dorsal view; the white arrow indicates the posterior margin of the distolateral projection; (**B**) distal region of the distolateral projection; white arrowheads indicate the teeth; black arrows indicate the petasma opening; (**C**) distolateral projection in ventral view; white arrowheads indicate the row of teeth of the distal region of the distolateral projection; the black arrow indicates that the carina of the proximal region of the distolateral projection is absent; (**D**) – Detailed ventral view of distolateral projection; white arrowheads indicate the row of teeth of the distal region of the distolateral projection; the black arrow indicates the carina of the proximal region of the distolateral projection is absent; (**E**) *Appendix masculina* in dorsal view; (**F**) *Appendix masculina* in dorsal view; white arrowheads indicate the spines; (**G**): *Appendix masculina* in ventral view; the black arrow indicates the row of spines of the posterior margin, the white arrow indicates the central convex region; (**H)** – *Appendix masculina* in ventral view; the black arrow indicates the row of spines of the posterior margin, the white arrow indicates the central convex region

#### **Holotype**

Mexico: Tabasco, 18°31′40.25″N – 93°19′43.95″W, 05/XI/2014, col. Ku, M.A.M., 1♂ (MZUSP 39351).

#### **Paratypes**

Mexico: Tabasco, 18°31′40.25″N – 93°19′43.95″W, 05/XI/2014, col. Ku, M.A.M., 5♂, 3♀ (CCDB 5461).

#### Additional material examined

USA: Isle Dernier, Louisiana, 16/XI/1992, col. Bauer R.T., 3♂ (CCDB 5394) – Puerto Rico: Mayagüez Bay, XII/1985, col. R.T. Bauer (CCLC 0417). Brazil: Amapá, Oiapoque, Estuário do Rio Oiapoque, Parna Cabo Orange, 04°22′17.6″N – 51°24′26.4″W, 22/VIII/2013, col. I.M. Vieira, A.G. Santiago & E.G. Oliveira, 1♀ (IEPA 1617) – Pará, Vigia, Ponta Seca, 0°51′45.00″S – 48°7′50.00″W, 19/XI/1994, col. M.P. Barros, 1♀ (MCP 2024).

#### Description

Petasma: In dorsal view, the proximal region occupies 3/4 of the distolateral projection length, posterior margins slightly rounded (Figs. 12B, 13A). The distal region extends for 1/4 of the distolateral projection length. The opening is wide and rounded, with a convex row of teeth (Figs. 12C, 13B). In ventral view, the row of teeth of the distal region of the posterior margin invades the proximal region and becomes sparser until it reaches its wider part (Fig. 13C,D). In this species the carina in the ventral surface of the proximal region is absent (Fig. 13C,D). The *appendix masculina* is subcircular but less rounded than in *X. kroyeri* (Fig. 13E,F) and has no prominent apex, but its posterior margin is covered with spines. The rounded projection is the widest of all species. In ventral view there are several rows of spines which cover the central convex region completely (Fig. 13G,H).

#### Etymology

The new species in named in the honour of our colleague Dr. Raymond Bauer, a recognized carcinologist who has devoted his career to the study of the biology of caridean shrimps, especially from Gulf of Mexico, the type locality of the new species herein described.

#### Type locality

Tabasco, Mexico.

#### Distribution

Mexico (Tabasco), USA (Louisiana, Texas); Puerto Rico, Brazil (Amapá, Pará).

#### Remarks

The individuals of our clade A3 correspond to a new species, undetected in previous studies. The shape of the petasma is very similar to that of *Xiphopenaeus dincao* nov. sp. The main character that differentiates these two species is the ventral surface of the *appendix masculina* covered with spines in *Xiphopenaeus baueri* nov. sp. The two species seem to occur in sympatry, at least in the north of Brazil, which may hamper their differentiation. In the Gulf of Mexico, however, *Xiphopenaeus baueri* nov. sp. is the only known species of *Xiphopenaeus*. Despite the vast list of works in the literature that studied biology of *X. kroyeri*, it was not possible to identify in which of these studies were used specimens of the described new species and thus the synonymic list was not provided.

### *Xiphopenaeus riveti* - P1

#### Material examined

Costa Rica: Puntarenas, Sierpe, Terraba, GPS coordinates unknown, VI/2013, J.S. Vargas, 4♂, 1♀ (CCDB 5247).

#### Description

**Figure 14:**
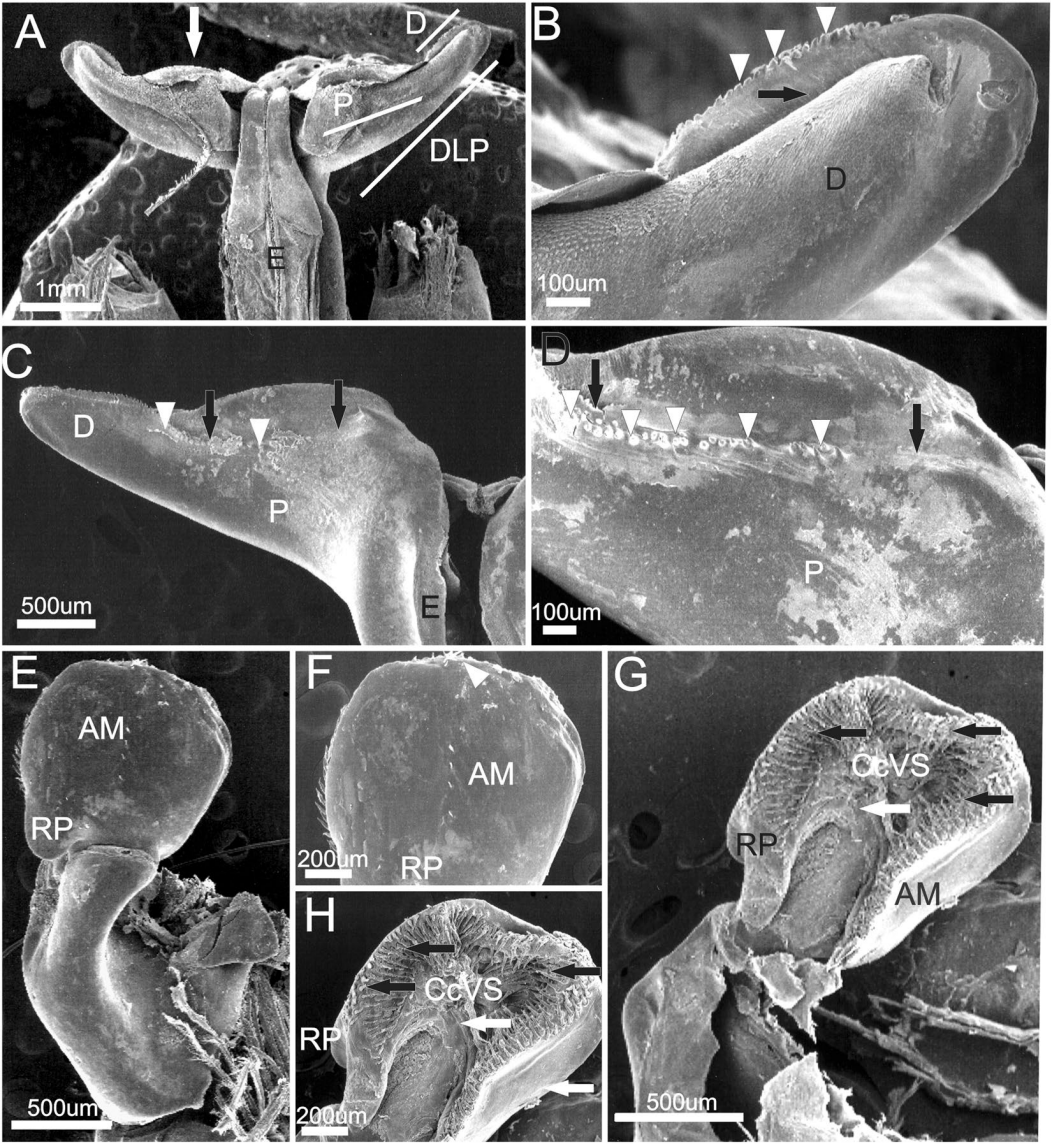
Scanning electron microscopy. Mexico: Tehuantepec, 4♂ (CCDB 5247). Male secondary sexual characters of *Xiphopenaeus riveti*. (**A**) petasma in dorsal view; the white arrow indicates the posterior margin of the distolateral projection; (**B**) distal region of the distolateral projection; white arrowheads indicate the teeth; black arrows indicate the petasma opening; (**C**) distolateral projection in ventral view; white arrowheads indicate the row of teeth of the distal region of the distolateral projection; the black arrow indicates the carina of the proximal region of the distolateral projection; (**D)** –detailed ventral view of distolateral projection; white arrowheads indicate the row of teeth of the distal region of the distolateral projection; the black arrow indicates the carina of the proximal region of the distolateral projection; (**E**) *Appendix masculina* in dorsal view; (**F**) *Appendix masculina* in dorsal view; (**G**) *Appendix masculina* in ventral view; the black arrow indicates the row of spines of the posterior margin, the white arrow indicates the central convex region; (**H)** – *Appendix masculina* in ventral view; the black arrow indicates the row of spines of the posterior margin, the white arrow indicates the central convex region.

Petasma: in dorsal view the proximal region occupies 2/3 of the distolateral projection length and the posterior margin is strongly rounded (Fig. 14A). Opening narrow and long with a row of teeth slightly rounded (Fig. 14B). In ventral view, the row of teeth of the posterior margin reaches 3/4 of the proximal region of the distolateral projection and goes through the margins of a well-defined carina that occupies the entire proximal region of the distolateral projection (Fig. 14C,D). *Appendix masculina* subcircular (Fig. 14E,F); the rounded projection is more elongated than in *X. kroyeri* but less than in *Xiphopenaeus baueri* nov. sp.; the posterior distal margin has few spines. In ventral view, the posterior margin is densely covered by long spines; the concave central region of the ventral surface lacks spines (Fig. 14G,H).

## Discussion

Our results revealed that the genus *Xiphopenaeus* is composed of at least five species, distributed along the American continental coasts. A division of the genus in three species, including *X. riveti* and a new species from the Atlantic, was suggested previously^9,10^, without a formal taxonomic and nomenclatural record and a detailed description. However, these studies were restricted to the South American coast and one locality from the Pacific. Thus, besides supporting the revalidation of *X. riveti* and a species from the Atlantic (*Xiphopenaeus dincao* nov. sp.), our study also indicates the existence of a third species from the Atlantic (*Xiphopenaeus baueri* nov. sp.) and at least two entities  from the Pacific. This last species (group P2) could not be described since only a female was available, and diagnostic morphological characters from the petasma are needed.

The use of molecular tools, in addition to the morphological ones, was extremely useful to strengthen and deepen our study, which confirms their usefulness in the taxonomic identification and to solve taxonomic and phylogenetic problems^23,26–30^. The between-group distances resulting from the phylogenetic analyses of COI and 16S were in the range of interspecific distances expected in decapods. Moreover, the genetic groups were supported by morphological differences in the male secondary sexual characters. Within the order Decapoda, the highest intraspecific distances known for the COI gene (barcoding region) are ~2%, while the interspecific distances between congeneric species are usually higher than 5%, reaching 10% in many cases, and even more than 30%^31,32^. For instance, in shrimps of the family Penaeidae from the coast of Egypt and in species of the genus *Farfantepenaeus*, the interspecific distances varied from 3 to 20%^33,34^. Thus, most interspecific distances detected here are above the threshold used to separate congeneric species based on the barcoding region.

The genetic distance between *Xiphopenaeus dincao* nov. sp. and *Xiphopenaeus baueri* nov. sp. for the COI gene are in between the intraspecific and interspecific thresholds of Decapoda. Even though intraspecific distances of up to 2.7% were reported for the penaeid shrimp *Artemesia longinaris* Spence Bate, 1888, it was less than 1% in other Atlantic Dendrobranchiata shrimps, such as *Pleoticus muelleri* (Spence Bate, 1888), *Farfantepenaeus paulensis* (Pérez-Farfante, 1967), *Farfantepenaeus brasiliensis* (Latreille, 1817), and *Farfantepenaeus subtilis* (Pérez-Farfante, 1967)^22,35–38^.

The intraspecific distances (0.0–0.2% in *Xiphopenaeus dincao* nov. sp.; 0.0–0.3% in *Xiphopenaeus baueri* nov. sp.) were much lower than the interspecific (2.7–3.3%), thus, there is a well-defined intraspecific gap. The DNA Barcoding technique uses this gap to identify the species^39–43^, and prove to be effective in the case of *Xiphopenaeus*.

The 16S gene is more conserved than the COI and has a low, or none intraspecific variation in decapod crustaceans. Moreover, in penaeid shrimps, the known interspecific distances between congeneric species are ~1%^44,45^. Nonetheless, our analysis of the 16S supported the genetic groups as distinct species, including the differentiation between *Xiphopenaeus dincao* nov. sp. and *Xiphopenaeus baueri* nov. sp.

The existence of a second species besides *X. riveti* in the Pacific (group P2) is an interesting and novel result since it has been undetected in previous studies. Unfortunately, we obtained DNA sequences only from a female from Tehuantepec, Mexico, warranting further descriptions. However, the genetic distances between P2 and the other Pacific specimens (P1), from Sierpe, Costa Rica, reinforce the idea that these two groups are indeed two distinct species. Further studies, with a more comprehensive sampling including more localities are needed to understand their geographic range in the Pacific. There is likely an overlap in the distribution range of these two species because the specimens from Panama, analysed by Gusmão *et al*.^9^, belong to our group P2, while the specimens from P1 came from Costa Rica, which is close to Panama. Since the holotype of *X. riveti* could not be located (it is probably lost), its comparison with specimens of these two groups was not possible.

**Figure 15:**
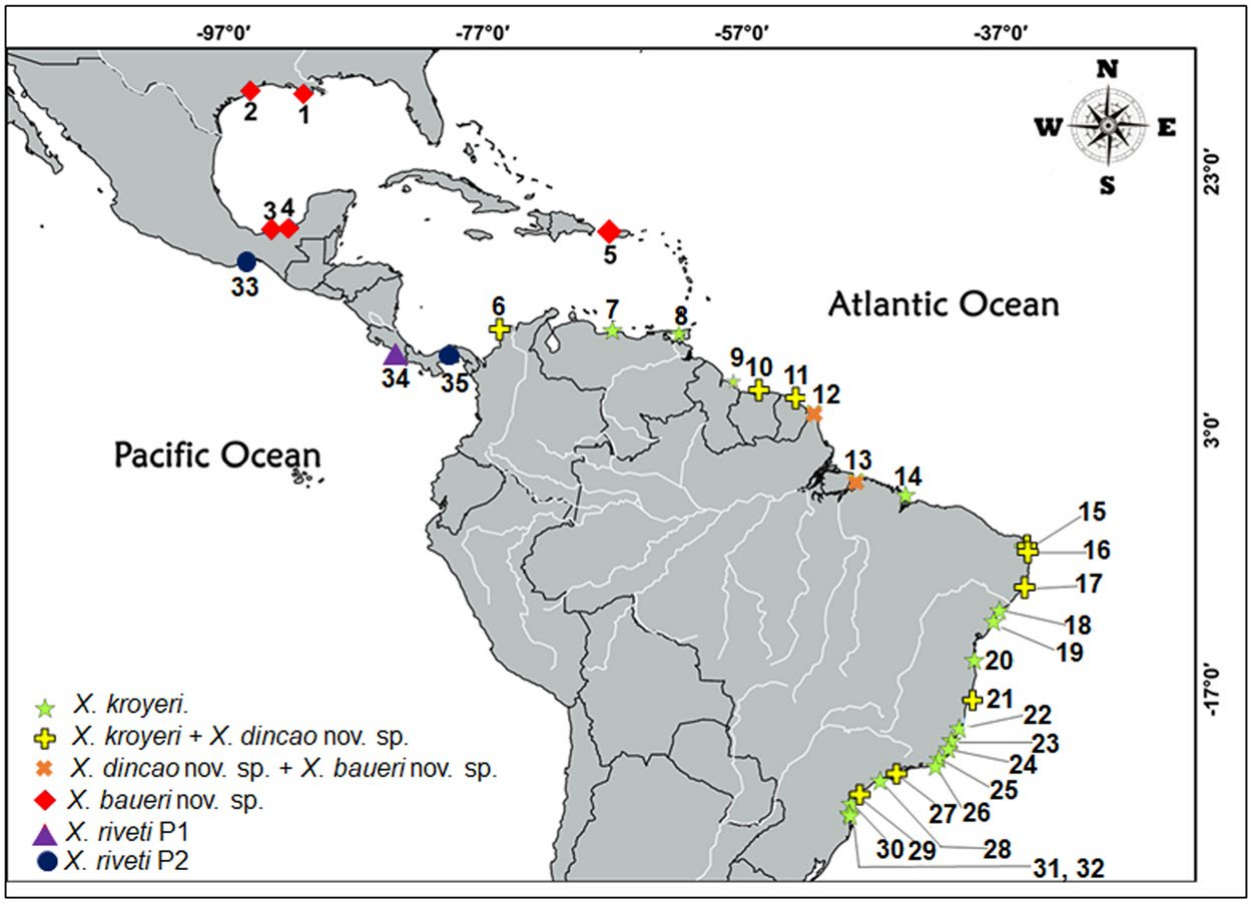
Geographical occurrence of the Atlantic *Xiphopenaeus* species. Locality numbers: 1- Isle Derniere (29°03′23.5″N; 90°48′58.6″W); 2- Galveston (29°11′49.3″N; 94°53′60.0″W); 3- Tabasco (18°31′40.25″N; 93°19′43.95); 4- Carmen (18°39′51.1″N; 91°51′17.7″W); 5- Mayaguez Bay (18°12′01.0″N; 67°09′37.0″W); 6- Colombia (); 7- Caracas (10°41′51.2″N; 66°56′41.2″W); 8- Trinidad and Tobago (10°24′28.8″N; 61°29′34.8″W); 9- Guyana (6°58′55.27″N; 57°54′31.07″W); 10- Suriname (5°56′35″N; 55°9′46″W); 11- French Guyana (4°54′44.698″N; 52°15′29.376″W); 12- Oiapoque (04°22′17.6″N; 51°24′26.4″W); 13- Vigia (00°51′45.00″S; 48°7′50.00″W); 14- São Luís (01° 59′ 56.7816″ S; 44° 19′ 7.8528″ W); 15- Natal (05°52′S; 35°10′W); 16- Baía Formosa (06°21′23.3″S; 35°00′24.7″W); 17- Maragogi (09°0′48.59″S; 35°13′14.46″W); 18- Aracaju (10°54′34″ S; 37°04′29″W); 19- Poças (11°46′S; 37°32 W); 20- Ilhéus (14°46′S; 39°01′W); 21- Caravelas (17°44′S; 39°15′W); 22- Nova Almeida (20°03′S; 40°11′W); 23- Marataízes (20°59′S; 40°47′W) 24- Atafona (21°37′33.3″S; 41°00′47.2″W); 25- Macaé (22°23.44′S; 41°44.57″W); 26- Arraial do Cabo (22°58′S; 42°01′W); 27- Ubatuba (23°26′S; 45°04′W); 28- Santos (23°58′S; 46°19′W); 29- Cananéia (25°02′S; 47°55′W); 30- Guaratuba (25°52′44.3778″ S; 48°31′35.7996″ W); 31- Barra Velha (26°37′S; 48°40′); 32- Balneário Camboriú (26°59′07″S; 48°35′58″W); 33- Tehuantepec (16°08′56.6″N; 95°09′12.1″W); 34- Sierpe (8°58′26.2″N; 83°37′58.9″W); 35- Panama City (8°53′N; 79°35′W).

An overlap between the geographical distribution of *X. kroyeri* and *Xiphopenaeus dincao* nov. sp. (as *Xiphopenaeus* sp. 1 and *Xiphopenaeus* sp. 2) along the Brazilian coast has been reported in Natal, Rio Grande do Norte and Ubatuba, São Paulo^9^, and later in Cananéia, São Paulo and in Caravelas, Bahia^10^. Here we show that they also overlap in Maragogi and Baía Formosa. *Xiphopenaeus dincao* nov. sp. and *Xiphopenaeus baueri* nov. sp. occurs in sympatry in Vigia and Oiapoque (Fig. 15). Considering that *X. kroyeri* has been collected in Caracas (Venezuela)^9^ and in São Luís, Maranhão (Brazil)^46^, the geographic distribution of these three species seem to overlap in the northern Atlantic coast of South America. In the present study, *Xiphopenaeus dincao* nov. sp. and *Xiphopenaeus baueri* nov. sp. were collected in the North of Brazil between Caracas and São Luís, Maranhão. Kerkhove *et al*.^11^ found *X. kroyeri* and *Xiphopenaeus dincao* nov. sp. (as *Xiphopenaeus* sp. 2) in Colombia and Guianan Marine Ecoregion. Therefore, based on our data, and on Gusmão *et al*.^9,46^, Piergiorge *et al*.^10^ and Kerkhove *et al*.^11^ the known geographical distribution of the species of the genus *Xiphopenaeus* is shown in Fig. 15.

It is likely that the successive openings and closures of the Panama Isthmus allowed the diversification of the genus *Xiphopenaeus* until its definitive closure circa 2.8 million years ago^47^. The low genetic distances seen between *Xiphopenaeus dincao* nov. sp. and *Xiphopenaeus baueri* nov. sp. suggest that these species diverged more recently. *Xiphopenaeus baueri* nov. sp. was the only species found in the Gulf of Mexico and probably originated there. The Quaternary sea level changes created or strengthened the isolation between this region and the Caribbean, warranting the exchange of migrants^48–51^. This isolation pattern has also been observed in other crustaceans such as the hermit crabs *Clibanarius vittatus* (Bosc, 1802) and *C. simetricus* (Randall, 1840), the first restricted to southeastern coast of the United States and Gulf of Mexico, and the second distributed along the Caribbean and South America^52^. Furthermore, in *Atya scabra* (Leach, 1816), individuals from the Gulf of Mexico are isolated from those from the Caribbean and Brazil, which share haplotypes among them although genetic divergences remain within the intraspecific level^53^.

The external genitalia of Dendrobranchiata display important characters for the taxonomy of the group and is useful to separate morphologically highly similar congeneric species^4,24,54^. Here, morphological differences supporting the separation of the groups identified in the molecular analyses were found in the male secondary sexual characters — petasma and *appendix masculina*. Previously, the petasma of *Xiphopenaeus* has been described without many details on the distolateral projections^4,55,56^. Fransozo *et al*.^57^, who studied the petasma development of *X. kroyeri* in Ubatuba, did not report morphological differences among the individuals. The photo provided by the authors corresponds to the shape described here to *Xiphopenaeus dincao* nov. sp. Burkenroad^6^ described a difference between individuals from Bahia (Brazil) and Louisiana (USA), concerning the “small tooth” of the distolateral projection, probably referring to the distal region of the distolateral projection. Considering the known geographical distribution of *Xiphopenaeus* (see Fig. 15), the species analysed by Burkenroad^6^ were probably *Xiphopenaeus kroyeri* stricto sensu and *Xiphopenaeus baueri* nov. sp.

We did not find any differences in the female external genitalia that could be used to separate the species of *Xiphopenaeus*. We expected that the same pattern revealed by the petasma would be seen in the thelycum, since the morphological differentiation of the reproductive structures contributes to the reproductive isolation and favours the formation of cryptic species^24^. Therefore, it seems that the hypothesis that the male and female genitalia evolve in a “key and lock” model as a mechanism of reproductive isolation^58^, does not apply to all Dendrobranchiata, as also reported in *Sicyonia* H. Milne Edwards, 1830^59^. Future studies on the ultrastructure of the internal part of the thelycum, including a larger number of specimens, are needed to solve the taxonomic issue based on females.

Previous description of the *appendix masculina* of *Xiphopenaeus* have been little informative. Smith^56^ described it as ovoid and flattened while Pérez-Farfante & Kensley^4^ described it as subcircular. Overall, the *appendix masculina* of *Xiphopenaeus* is similar to that of *Rimapenaeus fuscina*^60^, and may reflect the phylogenetic closeness between these genera^61,62^.

Microanatomic details may reveal crucial information to differentiate cryptic species^63^, and indeed, the use of SEM to analyse the petasma and *appendix masculina* revealed unknown details and ornamentations. Nonetheless, the gross anatomy of the petasma also allowed the identification of a few characters that were important to differentiate the species, without the need of such equipment (SEM). Thus, this information could facilitate and pave the way for further studies on the population biology and ecology of these cryptic species. According to Dall *et al*.^7^ the morphology of these structures are unique in each species. Using the stereomicroscope, it is possible to see the interspecific differences in the spines of the ventral face of the *appendix masculina*, which can also be enhanced using dyes like methyl blue.

To conclude, we demonstrate that the genus *Xiphopenaeus* is composed of at least five species, by combining morphological and molecular tools. Four out of five species can be differentiated based on the morphology, and two of them are described herein (*Xiphopenaeus dincao* nov. sp. and *Xiphopenaeus baueri* nov. sp.). Further studies addressing the status of the species from the Pacific and investigating the existence of more cryptic species are strongly encouraged.

## Methods

### Molecular analysis

We obtained specimens of the genus *Xiphopenaeus* from 17 localities: 15 from the Atlantic Ocean and two from the Pacific (Supplementary table S1 and S2). Most specimens came from the Crustacean Collection of the Department of Biology, FFCLRP, University of São Paulo, Brazil (CCDB). Others were collected during the development of research projects by members of the Laboratory of Marine and Freshwater Shrimps (LABCAM) and Laboratory of Bioecology ad Systematics of Crustaceans (LBSC). These specimens were stored in 80% ethanol and deposited in the collection mentioned above (CCDB) and Coleção de Crustáceos do Laboratório de Biologia de Camarões Marinhos e de Água Doce, Faculdade de Ciências, Universidade Estadual Paulista, Bauru, Brazil (CCLC). Additionally, we included specimens borrowed from the following scientific collections: Museu da Pontíficia Universidade Católica do Rio Grande do Sul (MCP); Instituto de Pesquisas Científicas e Tecnológicas do Estado do Amapá (IEPA), Colección Nacional de Crustáceos, Universidad Autónoma de Mexico (CNCR). We also received donations of specimens and tissues from the following scientific institutions: Zoological Collection of the University of Louisiana, Lafayette, USA (ULLZ) and Museo de Zoologia da Universidad de Costa Rica (MZ-UCR).

To extract DNA, muscle tissues were dissected from the abdomen and two common techniques were employed: the salting-out method^64^ with some modifications proposed by Mantelatto *et al*.^26^, and the Chelating Ion Exchange Resin (Chelex 100)^65^. Two molecular markers from mitochondrial genes, cytochrome c oxidase subunit I (COI) barcoding region^66–68^ (N = 91) and 16 S rDNA^67^ (N = 15) were used. Additionally, the Palumbi region of COI^25^ (N = 9) was used to allow the comparison of our specimens to those used by Gusmão *et al*.^9^ The amplification reactions contained bovine albumin 1% (Sigma), 10X Taq Buffer (Thermo Scientific), MgCl_2_ (25 mM), betaine (5 M), dNTPs (1.25 mM each), primers (10 or 20 µM) (Supplementary table S3), *Thermus aquaticus* polymerase (5 U µl^−1^) (Thermo Scientific), 1.0–5.5 µl of extracted DNA (50−100 ng ml^−1^), and distilled and deionized water to complete 25 µl.

The thermal cycler settings used for the amplification for COI were: 4 min at 94 °C for initial denaturation; 35 cycles of 30 s at 94 °C, 30 s at 40–46 °C, 60 s at 72 °C; and final extension for 10 min at 72 °C. For the 16 S they were: 4 min at 94 °C for initial denaturation; 40 cycles of 30 S at 48 °C; and final extension for 10 min at 72 °C. The results were checked in a 1.5% agarose gel stained with GelRed. Amplicons were purified with the SureClean Plus kit (Bioline USA Inc.) and sequenced using the BigDye Terminator Mix in an ABI 3730 XL DNA Analyzer (Applied Biosystems), following the manufacturer’s instructions.

Forward and reverse sequences were aligned to obtain the consensus sequence using BioEdit v.7.0.5^69^. Consensus sequences were aligned separately for each gene with the software MUSCLE (Multiple Sequence Comparison by Log-Expectation)^70^ on the online platform EMBL-EBI (European Molecular Biology Laboratory — The European Bioinformatics Institute)^71^ with the default parameters. The presence of stop codons in the COI sequences, which could indicate the occurrence of pseudogenes, was ruled out using the online translation tool EMBOSS Sixpack available in the EMBL-EBI Portal^72,73^.

### Phylogenetic analyses

The substitution saturation of all sequences was previously tested with the saturation test of Xia *et al*.^74^ using the software DAMBE. Highly variable regions were removed from the aligned sequences using Gblocks^75,76^ available online through the Castresana Lab, Animal Biodiversity and Evolution Program (http://molevol.cmima.csic.es/castresana/index.html).

The phylograms based on the Maximum Likelihood criterion were constructed in the program RAxML-HPC2 on X-SEDE^77^ available in the Cyber Infrastructure for Phylogenetic Research (CIPRES) website^78^. The default parameters of RAxML were used to perform the analysis for the GTR model. To measure the consistency of the topology we selected the option to automatically determine the number of bootstraps to be used in the RAxML^79^. Consequently, 1000 bootstrap pseudo-replicates were run, and only the values > 50% were reported.

### Genetic distances

Genetic distances were calculated with the Kimura 2-Parameter model^80^ based on COI (barcoding region) and 16S rDNA sequences separately, using MEGA 6.06^81^. We also included five other sequences of Dendrobranchiata species in our alignments to serve as external groups to rout the distance and phylogenetics analysis (Supplementary Table S4).

### Morphological assessment

The adult specimens used in the molecular analyses, and those from the biological collections CCDB and CCLC available for morphological analyses were carefully examined under a stereomicroscope. We searched for details in the characters and for morphological differences between the genetic groups indicated by the molecular analysis.

For the scanning electron microscopy (SEM), the petasma and *appendix masculina* were dissected from 15 voucher specimens preserved in 80% alcohol. These structures were dehydrated in a graded ethanol series of, 80%, 90%, and then placed 3x in 100% ethanol for 30 min. Afterwards, they were dried in a critical point dryer with liquid CO_2_ in an EMS 850 (Electron Microscopy Sciences) sputtering, properly placed on stubs with carbon adhesive tape and sputter-coated with gold (50 nm) in a Denton Vacuum Desk II sputtering. Micrographs were obtained in a Jeol JSM 5410 scanning electron microscope. Voucher specimens were deposited in CCDB (Catalog numbers — 5019, 5247) and in collection of Museu de Zoologia da Universidade de São Paulo (Catalog numbers — MZUSP 39350, MZUSP 39351). Ten samples containing two cryptic species were mixed and then checked using the morphological differences obtained from both stereomicroscopy and SEM. All samples were submitted again to the molecular protocol to validate the morphological characters.

## Supplementary information

supplemantary table

suplementary figures
