# Author Correction: A multigene and morphological analysis expands the diversity of the seabod shrimp *Xiphopenaeus* Smith, 1869 (Decapoda: Penaeidae), with descriptions of two new species

**DOI:** 10.1038/s41598-020-58157-6

**Published:** 2020-01-22

**Authors:** Abner Carvalho-Batista, Mariana Terossi, Fernando J. Zara, Fernando L. Mantelatto, Rogerio C. Costa

**Affiliations:** 1 Laboratório de Biologia de Camarões Marinhos e de Água Doce (LABCAM), Departamento de Ciências Biológicas, Faculdade de Ciências, Universidade Estadual Paulista (UNESP), Av. Luiz Edmundo Carrijo Coube 14-01, 17033-360 Bauru, SP Brazil; 2 Laboratório de Carcinologia, Departamento de Zoologia, Instituto de Biociências, Universidade Federal do Rio Grande do Sul (UFRGS), Av. Bento Gonçalves, 9500, Agronomia, 91501-970 Porto Alegre, Rio Grande do Sul Brazil; 3 Laboratório de Morfologia de Invertebrados (IML), Departamento de Biologia Aplicada, CAUNESP and IEAMar, Universidade Estadual Paulista (UNESP), FCAV. Via de Acesso Prof. Paulo Donato Castellane km 05, 14884-900 Jaboticabal, SP Brazil; 4 Laboratório de Bioecologia e Sistemática de Crustáceos (LBSC), Departamento de Biologia, Faculdade de Filosofia, Ciências e Letras de Ribeirão Preto (FFCLRP), Universidade de São Paulo (USP), Av. Bandeirantes 3900, 14040-901 Ribeirão Preto, SP Brazil

Correction to: *Scientific Reports* 10.1038/s41598-019-51484-3, published online 25 October 2019

This Article contains multiple typographical errors.

In the Results section, under the subheading ‘General description of petasma and *appendix masculina* of the genus *Xiphopenaeus’*:

“the central region of the ventral face is concave or convex and has rows of spines with varied distribution”

should read:

“the central ventral surface is concave or convex and has rows of spines with varied distribution”

In the legend of Figure 6:

“junction between the endopods with cincinuli (seta); C, *Xiphopenaeus kroyeri* (CCDB 5019): *appendix masculina* in dorsal view (DS – dorsal surface; RP – rounded projection); D, *Xiphopenaeus kroyeri* (CCDB 5019): *appendix masculina* in ventral view (VS - ventral surface; RP - rounded projection; white arrow indicating the row of spines of the posterior margin).

should read:

“junction between the endopods with cincinuli; C, *Xiphopenaeus kroyeri* (CCDB 5019): *appendix masculina* in dorsal view (DS – dorsal surface; RP – rounded projection); D, *Xiphopenaeus kroyeri* (CCDB 5019): *appendix masculina* in ventral view (VS - ventral surface; RP - rounded projection; white arrow indicating the central convex surface).

In the Results section, under the subheading ‘General description of petasma and *appendix masculina* of the genus Xiphopenaeus’:

“The posterior margin of the ventral surface is covered by small sparse spines, with a few rows in the central convex region”

should read:

“The posterior margin of the ventral surface is covered by small sparse spines, with a few rows in the central convex surface”

In the legend of Figure 8:

“*Appendix masculina* in ventral view; the black arrow indicates the row of spines of the posterior margin, white arrows indicate the convex central region; (H) *Appendix masculina* in ventral view; the black arrow indicates the row of spines of the posterior margin, white arrows indicate the convex central region”

should read:

“*Appendix masculina* in ventral view; the black arrow indicates the row of spines of the posterior margin, white arrows indicate the convex central surface; (H) *Appendix masculina* in ventral view; the black arrow indicates the row of spines of the posterior margin, white arrows indicate the convex central surface. (AM - *appendix masculina*; CxVS - central convex ventral surface; D - distal region of the distolateral projection; DLP - distolateral projection; E - endopod; P - proximal region of the distolateral projection; RP - round projection of *appendix masculina*).”

In the legend of Figures 11, 13 and 14:

“*Appendix masculina* in ventral view; the black arrow indicates the row of spines of the posterior margin, white arrows indicate the convex central region; (H) *Appendix masculina* in ventral view; the black arrow indicates the row of spines of the posterior margin, white arrows indicate the convex central region”

should read:

“*Appendix masculina* in ventral view; the black arrow indicates the row of spines of the posterior margin, white arrows indicate the convex central surface; (H) *Appendix masculina* in ventral view; the black arrow indicates the row of spines of the posterior margin, white arrows indicate the convex central surface. (AM - *appendix masculina*; CcVS - central concave ventral surface; D - distal region of the distolateral projection; DLP - distolateral projection; E - endopod; P - proximal region of the distolateral projection; RP - round projection of *appendix masculina*).”

In the legend of Figure 15:

“6-Colombia ()”

should read:

“6-Colombia (10°52′30″N; 74°24′10.800″W)”

In the results section, in the *Xiphopenaeus dincao* nov. sp. section under the subheading ‘Description’:

“The posterior margin of the ventral surface is covered by small sparse spines, with a few rows in the central convex region”

should read:

“The posterior margin of the ventral surface is covered by small sparse spines, with a few rows in the central concave surface”

In the Methods section, under the subheading ‘Morphological Assessment’:

“(Catalog numbers — 5019, 5247)”

should read:

“(5019, 5247, 5461, 6499)”

