# Supplementary material for: A multigene and morphological analysis expands the diversity of the seabod shrimp Xiphopenaeus Smith, 1869 (Decapoda: Penaeidae), with descriptions of two new species
Source: Sci Rep. 2019 Oct 25;9:15281. doi: 10.1038/s41598-019-51484-3 (PMC6814751; doi:10.1038/s41598-019-51484-3)
Supplement: Supplementary file 1 — supplemantary table [file 41598_2019_51484_MOESM1_ESM.docx]

**A multigene and morphological analysis expands the diversity of the seabod shrimp *Xiphopenaeus* Smith 1869 (Decapoda: Penaeidae), with descriptions of two new species**

**Abner Carvalho-Batista; Mariana Terossi; Fernando J. Zara; Fernando L. Mantelatto & Rogerio C. Costa**

**Supplementary Tables**

Supplementary Table S1. Specimens of the genus *Xiphopenaeus* used in the molecular analysis. CCDB: Coleção de Crustáceos do Departamento de Biologia da Faculdade de Filosofia, Ciências e Letras, Universidade de São Paulo, Ribeirão Preto, Brazil; CCLC: Coleção de Crustáceos do Laboratório de Biologia de Camarões Marinhos e de Água Doce, Faculdade de Ciências, Universidade Estadual Paulista, Bauru, Brazil; CNCR: Colección Nacional de Crustáceos, Universidad Autónoma, Mexico; IEPA: Instituto de Pesquisas Cientificas e Tecnológicas do Amapá, Brazil; MCP: Museu de Ciência e Tecnologia da Pontifícia Universidade Católica do Rio Grande do Sul, Brazil; ULLZ: Zoological Collection of the University of Louisiana, Lafayette, USA.

| **Ocean** | **Locality** | **Collection numbers** | **GenBank Accession numbers** | | |
| --- | --- | --- | --- | --- | --- |
|  |  |  | **COI Barcoding** | **COI Palumbi** | **16S** |
| Atlantic | Galveston, Texas, EUA | ULLZ 9900 | KY449152−154 | - | KY449075 |
|  | Tabasco, Mexico | CCDB 5461 | KY449141−149 | MH733312−13 | - |
|  | Carmen, Campeche, Mexico | CNCR 2830 | KY449150−051  MH737705−07 | - | - |
|  | Oiapóque, Amapá, Brazil | IEPA 1617 – 1618 | KY449136−137 | - | KY449072, KY449076 |
|  | Vigia, Pará, Brazil | MCP 2024 | KY449138−140 | - | KY449073 |
|  | Baía Formosa, Rio Grande do Norte, Brazil | CCDB 5337 | KY449091−099  MH727705−07 | - | KY449066 |
|  | Maragogi, Alagoas, Brazil | CCDB 5338, 5847, 6499 | KY449112−129 | - | KY449074 |
|  | Aracaju, Sergipe, Brazil | CCDB 5246 | KY449086 | - | KY449068 |
|  | Ilhéus, Bahia, Brazil | M20170004UESC | MH737703−04 | - | - |
|  | Marataízes, Espírito Santo, Brazil | CCDB 3985 | KY449103−106 | - | - |
|  | Macaé, Rio de Janeiro, Brazil | CCDB 5339 | KY449100−102 | - | KY449069 |
|  | Ubatuba, São Paulo, Brazil | CCDB 5018-5019 | KY449078−085 | - | KY449065 |
|  | Santos, São Paulo, Brazil | CCDB 3663 | KY449107−111 | MH737708−09 | - |
|  | Cananéia, São Paulo, Brazil | CCLC 0418 | KY449130−135 | MH737710−11 | - |
|  | Penha, Santa Catarina, Brazil | CCDB 5292 | KY449087−090 | - | KY449067 |
| Pacific | Tehuantepec, Oaxaca, Mexico | CCDB 0171 | KY449160 | MH737714 | KY449070 |
|  | Sierpe, Puntareñas, Costa Rica | CCDB 5247 | KY449155−159 | MH737716 | KY449071 |

Supplementary Table S2. Specimens of the genus *Xiphopenaeus* used in the molecular analysis and their sampling date (month and year). * Sampling which sympatry between *Xiphopenaeus kroyeri* and *Xiphopenaeus dincao* nov. sp. was detected. **Sampling which sympatry between *Xiphopenaeus dincao* nov. sp. and *Xiphopenaeus baueri* nov. sp. was detected.

| **Ocean** | **Locality** | **Collection numbers** | **Sampling date** |
| --- | --- | --- | --- |
| Atlantic | Galveston, Texas, EUA | ULLZ 9900 | Jun/2008 |
|  | Tabasco, Mexico | CCDB 5461 | Nov/2014 |
|  | Carmen, Campeche, Mexico | CNCR 2830 | Aug/1981 |
|  | Oiapóque, Amapá, Brazil | IEPA 1617,1618 | Aug/2013** |
|  | Vigia, Pará, Brazil | MCP 2024 | Nov/1994** |
|  | Baía Formosa, Rio Grande do Norte, Brazil | CCDB 5337 | Apr/2014* |
|  | Maragogi, Alagoas, Brazil | CCDB 5338, 6499 | Oct/2013* |
|  |  | CCDB 5847 | Jul/2015 |
|  | Aracaju, Sergipe, Brazil | CCDB 5246 | Jul/2013 |
|  | Ilhéus, Bahia, Brazil | M20170004UESC | Mar/2013 |
|  | Marataízes, Espírito Santo, Brazil | CCDB 3985 | Jun/2012 |
|  | Macaé, Rio de Janeiro, Brazil | CCDB 5339 | Jul/2014 |
|  | Ubatuba, São Paulo, Brazil | CCDB 5018, 5019 | Jul/2013 |
|  | Santos, São Paulo, Brazil | CCDB 3663 | Oct/2011 |
|  | Cananéia, São Paulo, Brazil | CCLC 0418 | Oct/2014 |
|  | Penha, Santa Catarina, Brazil | CCDB 5292 | May/2014 |
| Pacific | Tehuantepec, Oaxaca, Mexico | CCDB 0171 | Nov/06 |
|  | Sierpe, Puntareñas, Costa Rica | CCDB 5247 | Jun/2013 |

Supplementary Table S3. Primers used for gene amplification (COI: cytochrome c oxidase subunit I and 16S rDNA) by PCR (Polymerase Chain Reaction).

| **Gene** | **Primer** | **Sequence** | **Reference** |
| --- | --- | --- | --- |
| COI Barcoding Region | HCO1 | 5’-TAAACTTCAGGGTGACCAAAAAATCA-3’ | Folmer et al., 1994 |
|  | LCO1 | 5’-GGTCAACAAATCATAAAGATATTG-3’ |  |
|  | COH6 | 5´-TADACTTCDGGRTGDCCAAARAAYCA-3´ | Schubart & Huber, 2006 |
|  | COL6b | 5´- ACAAATCATAAAGATATYGG -3´ |  |
|  | COIAH2o | 5'-GACCAAAAAATCAGAATAAATGTTG -3' | Mantelatto et al., 2016 |
|  | COIAL2o | 5'-ACGCAACGATGATTATTTTCTAC-3' |  |
| COI  Palumbi  Region | COIa | AGT ATA AGC GTC TGG GTA GTC | Palumbi & Benzie, 1991 |
|  | COIf | CCT GCA GGA GGA GGA GAC CC |  |
| 16S | 16H9 | 5’-CCGGTCTGAACTCAGATCAC-3’ | Palumbi & Benzie, 1991 |
|  | 16L9 | 5’-CGCCTGTTTATCAAAAACAT-3’ |  |

Supplementary Table S4. Sequences of Dendrobranchiata species used as external groups to rout the genetic distance and phylogenetics analysis.

| Species | GenBank Accession Number | | |
| --- | --- | --- | --- |
|  | COI Barcoding | COI Palumbi | 16S |
| *Farfantepenaeus paulensis* (Pérez-Farfante, 1967) | KF783861 | - | KY449062 |
| *Farfantepenaeus brasiliensis* (Latreille, 1817) | KF783862 | - | KY449063 |
| *Rimapenaeus constrictus* (Stimpson, 1874) | KF783863 | - | KT959496 |
| *Belzebub faxoni* (Borradaile, 1915) | KY449077 | - | KY449064 |
| *Litopenaeus schmitti* (Burkenroad, 1938) | - | MH737717 | - |
