# Supplementary material for: A multigene and morphological analysis expands the diversity of the seabod shrimp Xiphopenaeus Smith, 1869 (Decapoda: Penaeidae), with descriptions of two new species
Source: Sci Rep. 2019 Oct 25;9:15281. doi: 10.1038/s41598-019-51484-3 (PMC6814751; doi:10.1038/s41598-019-51484-3)
Supplement: Supplementary file 2 — suplementary figures [file 41598_2019_51484_MOESM2_ESM.pdf]

A multigene and morphological analysis expands the diversity of the seabod shrimp *Xiphopenaeus* Smith, 1869 (Decapoda: Penaeidae), with descriptions of two new species

Abner Carvalho-Batista[1], Mariana Terossi[2], Fernando J. Zara[3], Fernando L. Mantelatto[4] & Rogerio C. Costa[1]

Supplementary Figures

Genetic groups	6	12	18	21	30	36	46	51	54	57	63	75	81	84	105	108	111	129	131	132	135	138	141	144	153	156	162
	A	A	C	C	A	T	C	C	G	C	T	T	A	T	C	C	T	C	T	G	T	G	T	G	A	C	A
	A	A	C	C	A	T	C	C	G	C	T	T	A	T	C	C	T	C	T	G	T	G	T	G	A	C	A
	A	A	C	C	A	T	C	C	G	C	T	T	A	T	C	C	T	C	T	G	T	G	C	G	A	C	A
	A	A	C	C	A	T	C	C	G	C	T	T	A	T	C	C	C	C	T	G	T	G	T	G	A	C	A
	A	A	C	C	A	T	C	C	G	C	T	T	A	T	C	C	T	C	G	G	T	G	T	G	A	C	A
	A	A	C	C	A	T	C	C	G	C	T	T	A	T	C	C	T	C	T	G	T	G	T	G	A	C	A
A1	A	A	C	C	A	T	C	C	G	C	T	T	A	T	C	C	T	C	T	G	T	G	T	G	A	C	A
	A	A	C	C	A	T	C	C	G	C	T	T	A	T	C	C	T	C	T	G	T	G	T	G	A	C	A
	A	A	C	C	A	T	C	C	G	C	T	T	A	T	C	C	T	C	T	G	T	G	T	G	A	C	A
	A	A	C	C	A	T	C	C	G	C	T	T	A	T	C	C	T	C	T	G	T	G	T	G	A	C	A
	A	A	C	C	A	T	C	C	G	C	T	C	A	T	C	C	T	C	T	G	T	G	T	G	A	C	A
	A	A	C	C	A	T	C	C	G	C	T	C	A	T	C	C	T	C	T	G	T	G	T	G	A	C	A
	A	A	C	C	A	T	C	C	G	C	T	C	A	T	C	C	T	C	T	G	T	G	T	G	A	C	A
	A	T	T	C	A	C	C	C	G	A	C	C	A	C	C	C	T	T	T	G	T	A	T	A	A	T	A
	A	T	T	C	A	C	C	C	G	A	C	C	A	C	C	C	T	T	T	G	T	A	T	A	A	T	A
A2	A	T	T	C	A	C	C	C	G	A	C	C	A	C	C	C	T	T	T	G	T	A	T	A	A	T	A
	A	T	T	C	A	C	C	C	G	C	C	C	A	C	C	C	T	T	T	G	T	A	T	A	A	T	A
	A	T	T	C	A	C	C	C	A	A	C	C	A	C	C	C	T	T	T	G	T	A	T	A	A	T	A
	A	T	C	C	A	C	C	C	G	A	C	C	A	C	C	C	T	T	T	A	T	A	T	A	A	T	G
	A	T	C	C	A	C	C	C	G	A	C	C	A	C	C	C	T	T	T	A	T	A	T	A	A	T	G
	A	T	C	C	A	C	C	C	G	A	C	C	A	C	C	C	T	T	T	A	T	A	T	A	A	T	G
A3	A	T	C	C	A	C	C	C	G	A	C	C	A	C	C	C	T	T	T	A	T	A	T	A	A	T	G
	A	T	C	C	A	C	C	C	G	A	C	C	A	C	C	C	T	T	T	A	T	A	T	A	A	T	G
	A	T	C	C	A	C	C	T	G	A	C	C	A	C	C	C	T	T	T	A	T	A	T	A	A	T	G
P1	G	C	C	T	G	C	T	C	A	A	T	C	A	T	T	T	T	T	T	A	C	A	T	A	A	C	A
	G	C	C	T	G	C	T	C	A	A	T	C	A	T	T	T	T	T	T	A	C	A	T	A	A	C	A
P2	A	T	T	T	G	C	T	C	A	A	C	C	G	T	T	T	T	T	T	A	T	G	T	A	G	T	A

(Column header spanning sites: Polymorphic nucleotide sites)

Supplementary figure S1. Polymorphic nucleotide sites of 27 Barcode COI haplotypes of the five genetic groups of *Xiphopenaeus* (continue).

Genetic groups	165	169	171	174	177	178	184	192	195	201	204	207	216	219	222	237	240	246	249	250	255	264	270	276
	T	C	A	C	T	T	C	C	A	A	T	C	A	C	T	C	A	C	T	T	C	A	C	G
	T	C	A	C	T	T	C	C	A	A	T	C	A	C	T	C	A	C	T	T	C	A	C	G
	T	C	A	C	T	T	C	C	A	A	T	C	A	C	T	C	A	C	T	T	C	A	C	G
	T	C	A	C	T	T	C	C	A	A	T	C	A	C	T	C	A	C	T	T	C	A	C	G
	T	C	A	C	T	T	C	C	A	A	T	C	A	C	T	C	A	C	T	T	C	A	C	G
	T	C	A	C	T	T	C	C	A	A	T	C	A	C	C	C	A	C	T	T	C	A	C	G
A1	T	C	A	C	T	T	C	C	A	A	T	C	A	C	T	C	A	C	T	T	C	A	C	G
	T	C	A	C	T	T	C	C	A	A	T	C	A	C	T	C	A	C	T	T	C	A	C	G
	T	C	A	C	T	T	C	C	A	A	T	C	A	C	T	C	A	C	T	T	C	A	C	G
	T	C	A	C	T	T	C	C	A	A	T	C	A	C	T	C	A	C	T	T	C	A	C	G
	T	C	A	C	T	T	C	C	A	A	T	C	A	C	T	C	A	C	T	T	C	A	C	G
	T	C	A	C	T	T	C	C	A	A	T	C	A	C	T	C	A	C	T	T	C	A	C	G
	T	C	A	C	T	T	C	C	A	A	T	C	A	C	T	C	A	C	T	T	C	A	C	G
	C	T	A	T	T	T	T	C	A	A	T	T	A	T	C	T	A	T	C	T	C	A	T	A
	C	T	A	T	T	T	T	C	A	A	T	T	A	T	C	T	A	T	T	T	C	A	T	A
A2	C	T	A	T	T	T	T	T	A	A	T	T	A	T	C	T	A	T	C	T	C	A	T	A
	C	T	A	T	T	T	T	C	A	A	T	T	A	T	C	T	A	T	C	T	C	A	T	A
	C	T	A	T	T	T	T	C	A	A	T	T	A	T	C	T	A	T	C	T	C	A	T	A
	C	T	A	T	T	T	T	C	T	G	T	T	G	C	C	T	C	T	C	T	C	A	T	A
	C	T	A	T	T	T	T	C	T	G	T	T	G	C	C	T	C	T	C	T	C	A	T	G
A3	C	T	A	T	T	T	T	C	T	A	T	T	G	C	C	T	C	T	C	T	C	A	T	A
	C	T	A	T	T	T	T	C	T	A	T	T	G	C	C	T	C	T	C	T	C	A	T	A
	C	T	A	T	T	T	T	C	T	A	T	T	G	C	C	T	C	T	C	T	C	A	T	A
	C	T	A	T	T	T	T	C	T	G	T	T	G	C	C	T	C	T	C	T	C	A	T	A
P1	T	T	A	T	T	C	T	A	A	A	T	C	A	C	T	C	T	T	C	C	T	G	T	A
	T	T	A	T	T	C	T	A	A	A	T	C	A	C	T	C	T	T	C	C	T	G	T	A
P2	T	C	G	T	A	C	T	A	A	A	C	C	A	C	T	C	T	T	C	C	C	G	C	A

(Column header spanning sites: Polymorphic nucleotide sites)

Supplementary figure S1. Polymorphic nucleotide sites of 27 Barcode COI haplotypes of the five genetic groups of *Xiphopenaeus* (continue).

Genetic groups	Polymorphic nucleotide sites 300	303	306	312	315	318	321	324	336	339	348	351	354	357	360	363	372	375	379	384	387	390	396	405
A1	T	A	C	T	C	T	A	G	C	C	C	C	A	T	A	A	C	C	C	A	A	C	G	A
	T	A	C	T	C	T	A	G	C	C	C	C	A	T	A	A	C	C	C	A	A	C	G	A
	T	A	C	T	C	T	A	G	C	C	C	C	A	T	A	A	T	C	C	A	A	C	G	A
	T	A	C	T	C	T	A	G	C	C	C	C	A	T	A	A	C	C	C	A	A	C	G	A
	T	A	C	T	C	T	A	G	C	C	C	C	A	T	A	A	C	C	C	A	A	C	G	A
	T	A	C	T	C	T	A	G	C	C	C	C	A	T	A	A	C	C	C	A	A	C	G	A
	T	A	C	T	C	T	A	G	C	C	C	T	A	T	A	A	C	C	C	A	A	C	G	A
	T	A	C	T	C	T	A	G	C	C	C	C	A	T	A	A	C	C	C	A	A	C	G	G
	T	A	C	T	C	T	A	G	C	C	C	C	A	T	A	A	C	C	C	A	A	C	G	A
	T	A	C	T	C	T	A	G	C	C	C	C	A	T	A	A	C	C	C	A	A	C	G	A
	T	A	C	T	C	T	A	G	C	C	C	C	A	T	A	A	C	C	C	A	A	C	G	A
	T	A	C	T	C	T	A	G	C	C	C	C	A	T	A	A	C	C	C	A	A	C	G	A
	T	A	C	T	C	T	A	G	C	C	C	C	A	T	A	A	C	C	C	A	A	C	G	A
A2	T	A	C	T	T	T	A	G	T	T	T	T	A	T	A	G	T	T	T	C	A	T	A	A
	T	A	C	T	T	T	A	G	T	T	T	T	A	T	A	G	T	T	T	C	A	T	A	A
	T	A	C	T	T	T	A	G	T	T	T	T	A	T	A	G	T	T	T	C	A	T	A	A
	T	A	C	T	T	T	A	G	T	T	T	T	A	T	A	G	T	T	T	C	A	T	A	A
	T	A	C	T	T	T	A	G	T	T	T	T	A	T	A	G	T	T	T	C	A	T	A	A
A3	T	G	C	T	T	T	A	G	T	T	T	T	A	T	G	T	T	T	T	C	A	T	A	A
	T	G	C	T	T	T	A	G	T	T	T	T	A	T	G	T	T	T	T	C	A	T	A	A
	T	G	C	T	T	T	A	G	T	T	T	T	A	T	G	T	T	T	T	C	A	T	A	A
	T	G	C	T	T	T	A	G	T	T	T	T	A	T	G	T	T	T	T	C	A	T	A	A
	T	G	C	T	T	T	A	G	T	T	T	T	A	T	G	T	T	T	T	C	A	T	A	A
	T	G	C	T	T	T	A	G	T	T	T	T	A	T	G	T	T	T	T	C	A	T	A	A
P1	T	A	A	T	T	C	A	A	T	T	C	G	A	T	G	G	G	A	T	T	G	T	A	G
	T	A	A	T	T	C	A	A	T	T	C	G	A	T	G	G	G	A	T	T	G	T	A	G
P2	C	A	A	C	T	T	G	T	T	T	C	G	G	C	A	G	T	C	T	T	A	T	A	A

Supplementary figure S1. Polymorphic nucleotide sites of 27 Barcode COI haplotypes of the five genetic groups of *Xiphopenaeus* (continue).

Genetic groups	Polymorphic nucleotide sites 408	417	432	435	441	444	448	450	462	465	467	471	472	474	477	480	486	495	501	516	520	528	529	534
A1	C	C	T	C	A	T	G	G	C	A	T	C	C	A	C	A	A	T	C	C	C	G	T	A
	C	C	T	C	A	T	G	G	C	A	T	C	C	A	C	A	A	T	C	C	C	G	T	A
	C	C	T	C	A	T	G	G	C	A	T	C	C	A	C	A	A	T	C	C	C	G	T	A
	C	C	T	C	A	T	G	G	C	A	T	C	C	A	C	A	A	T	C	C	C	G	T	A
	C	C	T	C	A	T	G	G	C	A	T	C	C	A	C	A	A	T	C	C	C	G	T	A
	C	C	T	C	A	T	G	G	C	A	T	C	C	A	C	A	A	T	C	C	C	G	T	A
	C	C	T	C	A	T	G	G	C	A	T	C	C	A	C	A	A	T	C	C	C	G	T	A
	C	C	T	C	A	T	G	G	C	A	T	C	C	A	C	A	A	T	C	C	C	G	T	A
	C	C	G	C	A	T	C	G	C	A	T	C	C	A	C	A	A	T	C	C	C	G	T	A
	C	C	T	C	A	T	G	G	C	A	C	C	C	A	C	A	A	T	C	C	C	G	T	A
	C	C	T	C	A	T	G	G	C	A	T	C	C	A	C	A	A	T	C	C	C	G	T	A
	C	C	T	C	A	T	G	G	C	A	T	C	C	A	C	A	A	T	C	C	C	G	T	A
	C	C	T	C	A	T	G	G	C	A	T	C	C	A	C	A	A	T	C	C	C	G	T	A
A2	C	T	T	T	A	C	G	A	C	A	T	A	T	G	T	T	A	C	T	T	T	G	T	A
	C	T	T	T	A	C	G	A	C	A	T	A	T	G	T	T	A	C	T	T	T	G	T	A
	C	T	T	T	A	C	G	A	C	A	T	A	T	G	T	T	A	C	T	T	T	G	T	A
	C	T	T	T	A	C	G	A	C	A	T	A	T	G	T	T	A	C	T	T	T	G	T	A
	C	T	T	T	A	C	G	A	C	A	T	A	T	G	T	T	A	C	T	T	T	A	T	A
A3	T	T	T	T	G	C	G	A	T	A	T	A	T	G	T	T	A	C	T	T	T	A	T	G
	T	T	T	T	G	C	G	A	T	A	T	A	T	G	T	T	A	C	T	T	T	A	T	G
	T	T	T	T	G	C	G	A	T	A	T	A	T	G	T	T	A	C	T	T	T	A	T	G
	T	T	T	T	G	C	G	A	T	A	T	A	T	G	T	T	A	C	T	T	T	A	T	G
	T	T	T	T	G	C	G	A	T	A	T	A	T	G	T	T	A	C	T	T	T	A	T	G
	T	T	T	T	G	C	G	A	T	A	T	A	T	G	T	T	A	C	T	T	T	A	T	G
P1	C	T	T	T	A	C	G	A	C	A	T	A	T	A	T	A	G	T	C	T	T	G	C	G
	C	T	T	T	A	C	G	A	C	A	T	A	T	A	T	A	G	T	C	T	T	G	C	G
P2	C	T	T	T	A	G	G	A	C	G	T	A	T	A	T	G	A	C	C	T	T	G	T	A

Supplementary figure S1. Polymorphic nucleotide sites of 27 Barcode COI haplotypes of the five genetic groups of *Xiphopenaeus* (continue).

Genetic groups	Polymorphic nucleotide sites										
	546	549	558	561	568	573	582	585	588	591	594
A1	A	A	T	C	T	C	C	C	C	C	A
	A	A	T	C	T	C	C	C	C	T	A
	A	A	T	C	T	C	C	C	C	T	A
	A	A	T	C	T	C	C	C	C	T	A
	A	A	T	C	T	C	C	C	C	C	A
	A	A	T	C	T	C	C	C	C	C	A
	A	A	T	C	T	C	C	C	C	T	A
	A	A	T	C	T	C	C	C	C	C	A
	A	A	T	C	T	C	C	C	C	C	A
	A	A	T	C	T	C	C	C	C	C	A
	A	A	T	C	T	C	C	C	C	T	A
	A	A	T	C	T	C	C	C	C	C	A
	A	A	A	C	T	C	C	C	C	T	A
A2	T	A	A	T	T	T	T	T	T	A	A
	T	A	A	T	T	T	T	T	T	A	A
	T	A	A	T	T	T	T	T	T	A	A
	T	A	A	T	T	T	T	T	T	A	A
	T	A	A	T	T	T	T	T	T	A	A
A3	T	G	A	T	T	T	T	T	T	A	A
	T	G	A	T	T	T	T	T	T	A	A
	T	G	A	T	T	T	T	T	T	A	A
	T	G	A	T	T	T	T	T	T	A	A
	T	G	A	T	T	T	T	T	T	A	A
	T	G	A	T	T	T	T	T	T	A	A
P1	T	G	A	T	T	C	T	T	T	T	G
	T	G	A	T	T	T	T	T	T	T	G
P2	T	G	A	T	C	T	T	T	C	T	G

Supplementary figure S1. Polymorphic nucleotide sites of 27 Barcode COI haplotypes of the five genetic groups of *Xiphopenaeus*.

Genetic groups	Polymorphic nucleotide sites																		
	99	180	187	192	193	194	247	254	284	286	289	296	328	370	377	378	481	483	510
A1	T	A	C	A	C	A	A	T	C	T	C	A	C	C	T	T	T	A	T
A2	C	A	T	A	T	A	G	C	A	T	T	A	C	T	C	C	C	A	T
A2	C	A	T	A	T	A	G	C	A	T	T	A	C	T	C	C	C	A	T
A2	C	A	T	A	T	A	G	C	A	T	T	A	C	T	C	C	C	A	T
A3	C	A	T	C	T	A	G	T	A	T	C	T	T	T	C	C	C	G	T
A3	C	A	T	C	T	A	G	T	A	T	C	T	C	T	C	C	C	G	T
P1	C	A	C	A	C	A	A	T	A	C	T	A	C	C	T	T	T	A	C
P2	T	G	C	A	C	G	A	T	A	C	C	A	C	C	T	T	T	A	T

Supplementary figure S2. Polymorphic nucleotide sites of eight 16S haplotypes of the five genetic groups of *Xiphopenaeus.*

Genetic groups	Polymorphic nucleotide sites																					
	4	7	19	25	28	37	40	43	46	55	58	61	64	76	79	82	85	86	94	100	109	121	124
	C	C	G	C	T	C	A	C	C	A	A	A	T	C	T	A	C	C	G	C	T	C	T
	C	C	G	C	T	C	A	C	C	A	A	A	T	C	T	A	C	C	G	C	T	C	T
	C	C	G	C	T	C	A	C	C	A	A	A	T	C	T	A	C	C	G	C	T	C	T
Xiphopenaeus sp. 1	C	C	G	C	T	C	A	C	C	A	A	A	T	C	T	A	C	C	G	C	T	C	T
	C	C	G	C	T	C	A	C	C	A	A	A	T	C	T	A	C	C	G	C	T	C	T
	C	C	G	C	T	C	A	C	C	A	A	A	T	C	T	A	C	C	G	C	T	C	T
	C	C	G	C	T	C	A	C	T	A	A	A	T	C	T	A	C	C	G	C	T	C	T
A1	C	C	G	C	T	C	A	C	C	A	A	A	T	C	T	A	C	C	G	C	T	C	T
Xiphopenaeus sp. 2	C	C	C	T	T	T	G	T	T	A	G	G	T	T	C	A	T	T	G	C	C	T	A
	C	C	C	T	T	T	G	T	T	A	G	G	T	T	C	A	T	T	G	C	C	T	A
A2	C	C	C	T	T	T	G	T	T	A	G	G	T	T	C	A	T	T	G	C	C	T	A
A3	C	C	C	T	T	T	G	C	C	A	G	G	T	T	C	G	C	T	G	T	C	T	G
P1	A	T	T	T	C	T	A	T	C	G	A	A	T	T	C	A	T	T	A	C	C	C	A
Xiphopenaeus riveti	A	C	T	T	C	T	A	C	C	A	G	G	C	T	C	G	T	T	A	C	T	T	A
P2	-	-	-	-	-	-	-	-	-	A	G	G	C	T	C	G	T	T	A	C	T	T	A

Supplementary figure S3. Polymorphic nucleotide sites of 15 COI Palumbi region haplotypes of *Xiphopenaeus* including the specimens from our study assigned to the five genetic groups and the sequences from Gusmão et al. (2006) (continue).

Genetic groups	Polymorphic nucleotide sites																					
	127	133	136	139	142	145	151	157	163	166	172	175	181	187	199	208	223	226	235	238	245	247	250
	A	A	G	A	T	C	A	T	A	A	C	C	A	C	T	C	A	T	T	C	C	A	T
	A	A	G	A	T	C	A	T	A	A	C	C	A	C	T	C	A	T	T	C	C	A	T
	A	A	G	A	T	C	A	T	A	A	C	C	A	C	T	C	A	T	T	C	C	A	T
Xiphopenaeus sp. 1	A	A	G	A	T	C	A	T	A	A	C	C	A	C	T	C	A	T	T	C	C	A	T
	A	A	G	A	T	C	A	T	A	A	C	C	A	C	T	C	A	T	T	C	C	A	T
	A	A	G	A	T	C	A	T	A	A	C	C	A	C	T	C	A	T	T	C	C	A	T
	A	A	G	A	T	C	A	T	A	A	C	C	A	C	T	C	A	T	T	C	C	A	T
A1	A	A	G	A	T	C	A	T	A	A	C	C	A	C	T	C	A	T	T	C	C	A	T
Xiphopenaeus sp. 2	G	A	C	A	T	T	A	T	T	G	C	T	A	T	A	T	G	C	T	T	C	G	C
	G	A	C	A	T	T	A	T	T	G	C	T	A	T	A	T	G	C	T	T	C	G	C
A2	G	A	C	A	T	T	A	T	T	G	C	T	A	T	A	T	G	C	T	T	C	G	C
A3	G	A	T	A	T	T	A	T	T	A	C	C	A	T	A	T	G	G	T	T	T	A	T
P1	G	A	G	G	T	T	A	C	A	A	T	T	A	C	A	T	A	T	T	T	T	A	T
Xiphopenaeus riveti	A	G	A	A	C	T	G	C	A	A	T	T	A	C	A	T	A	T	C	T	T	A	C
P2	A	G	A	A	C	T	G	C	A	A	T	T	G	C	A	T	A	T	C	T	T	A	C

Supplementary figure S3. Polymorphic nucleotide sites of 15 COI Palumbi region haplotypes of *Xiphopenaeus* including the specimens from our study assigned to the five genetic groups and the sequences from Gusmão et al. (2006) (continue).

Genetic groups	Polymorphic nucleotide sites																					
	256	259	265	268	269	274	277	280	283	289	292	295	298	301	304	313	314	319	322	332	334	349	352
	C	C	C	A	C	C	C	T	T	C	A	G	C	C	A	C	C	T	A	C	A	T	T
	T	C	C	A	C	C	C	T	T	C	A	G	C	C	A	C	C	T	A	C	A	T	T
	C	C	C	A	C	C	C	T	T	C	G	G	C	C	A	C	C	T	A	C	A	T	T
Xiphopenaeus sp. 1	C	C	C	A	C	C	C	T	T	C	A	G	C	C	A	C	C	T	A	C	A	T	T
	C	C	C	A	C	C	T	T	T	C	A	G	C	T	A	C	C	T	A	C	A	T	T
	C	C	C	A	C	C	C	T	T	C	A	G	C	T	A	C	C	T	A	C	A	T	T
	C	C	C	A	C	C	C	T	T	C	A	G	C	C	A	C	C	T	A	C	A	T	T
A1	C	C	C	A	C	C	C	T	T	C	A	G	C	C	A	C	C	T	A	C	A	T	T
Xiphopenaeus sp. 2	C	T	C	G	T	T	C	T	C	T	A	A	T	T	G	T	T	T	T	C	A	T	A
	C	T	C	G	T	T	C	T	C	T	A	A	T	T	G	T	T	T	T	C	A	T	A
A2	C	T	C	G	T	T	C	T	C	T	A	A	T	T	G	T	T	T	T	C	A	T	A
A3	C	T	C	G	T	T	C	C	C	T	A	G	T	T	A	T	T	T	T	C	A	T	A
P1	T	C	T	A	T	C	C	T	C	T	A	G	C	T	A	C	T	T	T	C	G	T	G
Xiphopenaeus riveti	C	T	T	G	T	T	C	T	T	T	A	A	T	C	G	C	T	C	T	T	A	C	A
P2	C	T	T	G	T	T	C	T	T	T	A	A	T	C	G	C	T	C	T	T	A	C	A

Supplementary figure S3. Polymorphic nucleotide sites of 15 COI Palumbi region haplotypes of *Xiphopenaeus* including the specimens from our study assigned to the five genetic groups and the sequences from Gusmão et al. (2006) (continue).

Genetic groups	Polymorphic nucleotide sites																						
	355	358	361	367	370	373	374	379	382	388	391	394	400	409	415	416	421	433	442	445	448	451	452
	C	T	A	C	T	C	C	C	C	C	C	A	T	C	C	T	G	A	T	C	C	A	A
	C	T	A	C	T	C	C	C	C	C	C	A	T	C	C	T	G	A	T	C	C	A	A
	C	T	A	C	T	C	C	C	C	C	C	A	T	C	C	T	G	A	T	C	C	A	A
Xiphopenaeus sp. 1	C	T	A	C	T	C	C	C	C	C	C	A	T	C	C	T	G	A	T	C	C	A	A
	C	T	A	C	T	C	C	C	C	C	C	A	T	C	C	T	A	A	T	C	C	A	A
	C	T	A	C	T	C	C	C	C	C	C	A	T	C	C	T	A	A	T	C	C	A	A
	C	T	A	C	T	C	C	C	C	C	C	A	T	C	C	T	G	A	T	C	C	A	A
A1	C	T	A	C	T	C	C	C	C	C	C	A	T	C	C	T	G	A	T	C	C	A	A
Xiphopenaeus sp. 2	T	T	A	C	T	T	T	C	T	C	T	G	T	C	T	T	A	C	C	T	C	A	G
	T	C	A	C	T	T	T	C	T	C	T	G	T	C	T	T	A	C	C	T	C	A	G
A2	T	T	A	C	T	T	T	C	T	C	T	G	T	C	T	T	A	C	C	T	C	A	G
A3	T	T	A	C	T	T	T	C	T	T	T	G	T	T	T	T	A	A	C	T	T	A	G
P1	C	C	A	T	T	T	T	C	C	C	C	A	C	C	T	C	C	T	C	T	T	G	G
Xiphopenaeus riveti	T	C	G	T	C	T	T	T	C	C	C	A	C	C	C	T	C	T	C	C	T	A	G
P2	T	C	G	T	C	T	T	T	C	C	C	A	C	C	C	T	C	T	C	C	T	A	G

Supplementary figure S3. Polymorphic nucleotide sites of 15 COI Palumbi region haplotypes of *Xiphopenaeus* including the specimens from our study assigned to the five genetic groups and the sequences from Gusmão et al. (2006) (continue).

Genetic groups	Polymorphic nucleotide sites																						
	460	466	469	475	481	484	488	490	493	511	514	517	520	529	532	538	541	542	544	551	553	556	559
	T	T	T	T	C	T	C	C	C	C	C	C	T	T	A	A	C	A	C	T	T	C	A
	T	T	T	T	C	T	C	C	C	C	C	C	T	T	A	A	C	A	C	T	T	C	A
	T	T	T	T	C	T	C	C	C	C	C	C	T	T	A	A	C	A	C	T	T	C	A
Xiphopenaeus sp. 1	T	T	T	T	C	T	C	C	C	C	C	C	T	T	A	A	C	A	C	T	T	C	A
	T	T	T	T	C	T	C	C	C	C	C	C	T	T	A	A	C	A	C	T	T	C	A
	T	T	T	T	C	T	C	C	C	C	C	C	T	T	A	A	C	A	C	T	T	C	A
	T	T	T	T	C	T	C	C	C	C	C	C	T	T	A	A	C	A	C	T	T	C	A
A1	T	T	T	T	C	T	C	C	C	C	C	C	T	T	A	A	C	A	C	T	T	C	A
Xiphopenaeus sp. 2	T	C	T	C	T	T	C	T	T	T	T	C	C	C	A	A	T	G	C	T	C	T	A
	T	C	T	C	T	T	C	T	T	T	T	C	C	C	A	A	T	G	C	T	C	T	A
A2	T	C	T	C	T	T	C	T	T	T	T	C	C	C	A	A	T	G	C	T	C	T	A
A3	T	C	T	C	C	T	C	A	T	T	C	C	C	T	A	T	T	G	C	T	C	T	A
P1	C	T	C	T	C	T	T	A	C	T	C	C	T	T	A	A	T	G	T	T	C	T	A
Xiphopenaeus riveti	T	T	T	T	C	C	C	A	C	C	C	T	T	C	G	A	T	A	C	C	C	T	G
P2	T	T	T	T	C	C	C	A	C	C	C	T	-	-	-	-	-	-	-	-	-	-	-

Supplementary figure S3. Polymorphic nucleotide sites of 15 COI Palumbi region haplotypes of *Xiphopenaeus* including the specimens from our study assigned to the five genetic groups and the sequences from Gusmão et al. (2006) (continue).

Genetic groups	Polymorphic nucleotide sites								
	562	565	568	571	572	580	583	589	592
	T	C	G	T	T	A	G	C	C
	T	C	G	C	T	A	G	C	C
	T	C	G	T	T	A	G	C	C
Xiphopenaeus sp. 1	T	C	A	T	T	A	G	C	C
	T	C	G	C	T	A	G	C	C
	T	C	G	C	T	A	G	C	C
	T	C	G	T	T	A	G	C	C
A1	T	C	G	T	T	A	G	C	A
Xiphopenaeus sp. 2	C	T	A	T	T	T	A	T	C
	C	T	A	T	T	T	A	T	C
A2	C	T	A	T	T	T	-	-	-
A3	C	T	A	T	T	T	A	T	A
P1	C	T	A	T	T	T	A	T	A
Xiphopenaeus riveti	C	T	A	T	C	T	A	T	C
P2	-	-	-	-	-	-	-	-	-

Supplementary figure S3. Polymorphic nucleotide sites of 15 COI Palumbi region haplotypes of *Xiphopenaeus* including the specimens from our study assigned to the five genetic groups and the sequences from Gusmão et al. (2006).
